# [Au_4_(dppb)_3_]^2+^ – A Planar Gold Cluster with Bright Excitation Wavelength Dependent Emission

**DOI:** 10.1002/smll.75130

**Published:** 2026-08-18

**Authors:** Markus Strienz, Jakob Keck, Wolfgang Leis, Jonas L. Hiller, Claudio Schrenk, Christoph Kertzscher, Michael Seitz, Marcus Scheele, Alfred J. Meixner, Andreas Schnepf

**Affiliations:** ^1^ Institute of Inorganic Chemistry University of Tübingen Tübingen Germany; ^2^ Institute of Physical and Theoretical Chemistry University of Tübingen Tübingen Germany

**Keywords:** cluster compounds, excitation dependent emission, gold, laser spectroscopy, luminescence, phosphane ligands, single crystal

## Abstract

The reaction of dppb(AuCl)_2_ and InCl gives the [Au_4_(dppb)_3_]^2+^ cluster where the four gold atoms are arranged in a deltoid. This contrasts all known [Au_4_]^2+^ clusters which exclusively form tetrahedral structures. Single crystals of the title compound luminesce with a high quantum yield in the near infrared at room temperature. The excitation wavelength dictates the emission wavelength which ranges from 680 to 918 nm at 77 K. Comprehensive studies of the excitation‐dependent emission and luminescence lifetimes in both polycrystalline and single‐crystalline samples provide a unique and detailed optical profile of the cluster, uncovering distinct photophysical behavior central to its emissive properties.

## Graphical abstract

The first planar [Au_4_]^2+^ cluster with a deltoid geometry displays red to near‐infrared emission tunable from 700 to 918 nm. A quantum yield of 17% is observed at 770 nm upon 375 nm excitation. Excitation‐wavelength‐dependent shifts in emission reveal the involvement of multiple excited states, highlighting unprecedented photophysical versatility in gold clusters.

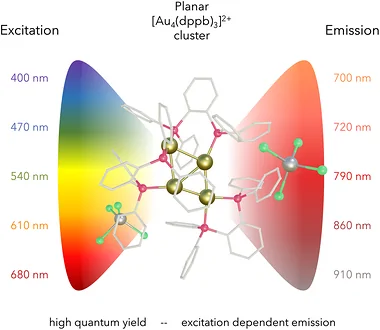

## 1 Introduction

Gold clusters have attracted increasing attention due to their model system character for the transition between metal atoms and the bulk, with a plethora of different structures and forms [1, 2, 3]. Due to their unique structures and the resulting novel properties, gold clusters have found applications in catalysis, medicine, electronics and optics [4, 5, 6, 7]. A major advantage of clusters in comparison to e.g. nanoparticles is their atomic precision, allowing crystallization and exact determination of the position of the atoms and the intra‐ and intermolecular distances. Such information is essential for the understanding of the above‐mentioned applications and for further development and improvement.

The tetrahedral motif is predominantly present in small gold clusters, and most clearly seen in the tetrahedral structure of all [Au_4_]^2+^ clusters [8, 9]. However, this is not the case for [Au_4_]^4+^ compounds, which exhibit square and chain‐like structures with no direct gold–gold contacts (for a more detailed comparison see Table S2) [10, 11, 12, 13]. The proximity between the gold atoms in such Au(I)_x_ complexes (x > 1) is primarily due to bidentate ligands and, to a lesser extent, to aurophilic interactions [10, 11, 12, 13].

Whereas most gold clusters, particularly larger ones with a polyhedral arrangement of the gold atoms, exhibit only negligible luminescence [1], Au(I) compounds are characterized by intense emission with long lifetimes, combined with high stability and excellent synthetic yields [14, 15]. Among other fascinating optical properties, metal complexes and clusters might exhibit excitation dependent emission properties, where the emission wavelength of these complexes shifts with the excitation wavelength as recently shown [16, 17, 18]. In this study, we present the first [Au_4_]^2+^ cluster that possesses no tetrahedral structure. Furthermore, this compound represents the first gold cluster with visible excitation‐dependent emission in the crystalline state, allowing a more detailed study of this phenomenon. We show that alongside the emission spectrum, the excited state lifetime also depends strongly on the excitation energy. Combining the crystal structure and Density Functional Theory (DFT) calculations with spectroscopic analysis, we propose that the excitation‐dependent emission is due to the formation of different excited states within the cluster.

## 2 Results and Discussion

The reaction of dppb(AuCl)_2_ (dppb = 1,2‐bis(diphenylphosphino)benzene) with InCl gives [Au_4_(dppb)_3_][InCl_4_][InCl_4_(THF)] (**1**), crystallizing with THF in form of yellow needles in the monoclinic space group *P*2_1_/*c* (Figure 1a). The crystallization from a diethyl ether/tetrahydrofuran solution gives 50% yield within one week.

**FIGURE 1:**
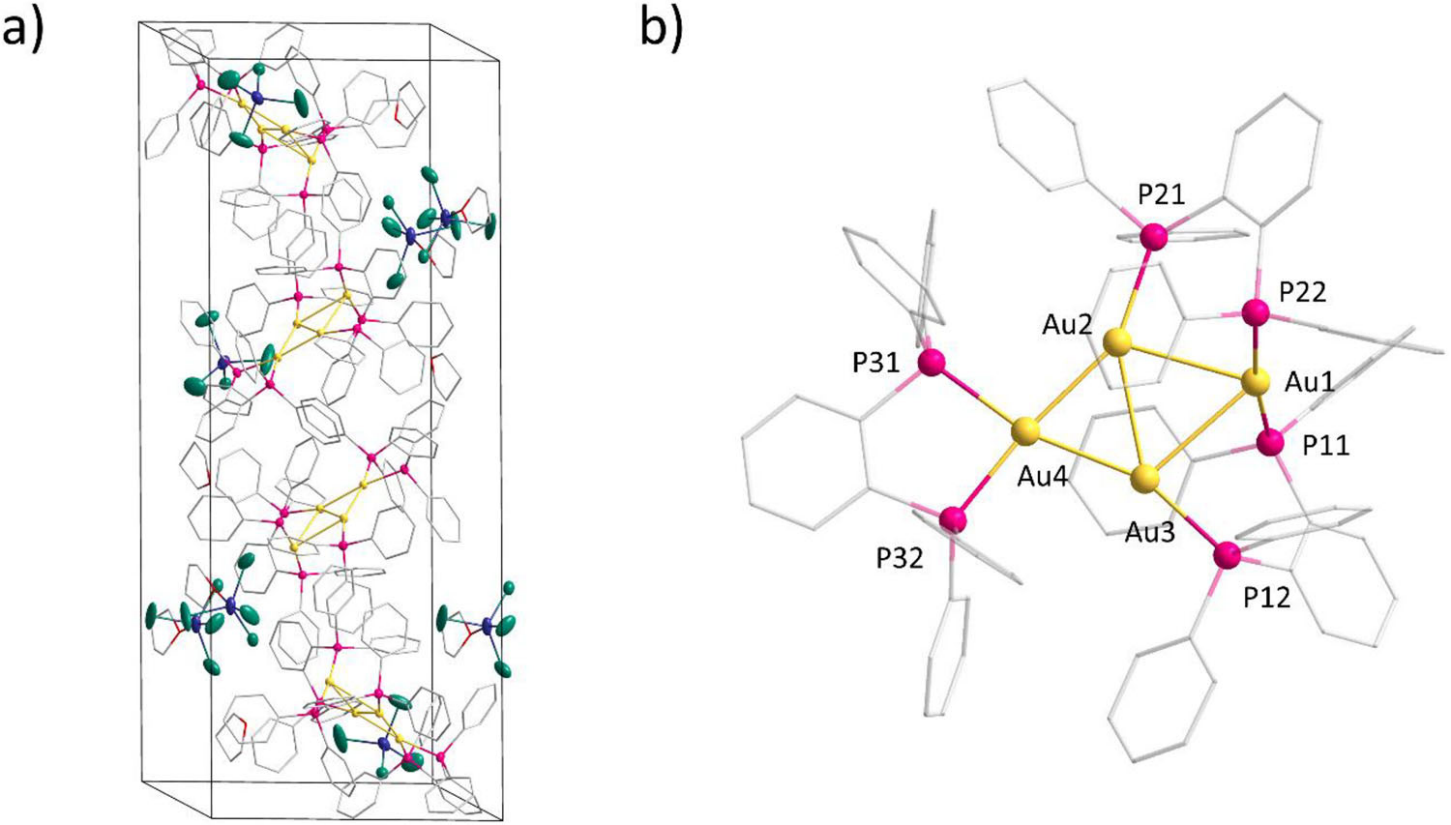
(a) Unit cell of **1** in the solid state. All atoms except for carbon are displayed as thermal ellipsoids with 50% probability. (b) Ball and stick structure of [Au_4_(dppb)_3_]^2+^ in the solid state. Hydrogen atoms are omitted for clarity. Au: yellow, In: blue, P: pink, Cl: green, C: grey.

The title compound **1** is a salt, with a dicationic [Au_4_(dppb)_3_]^2+^ cluster as the cation and the two anions [InCl_4_]^−^ and [InCl_4_(THF)]^−^. Thereby one of the two indium atoms is coordinated by a THF molecule. The cation consists of four gold atoms, ordered in form of a distorted deltoid (Figure 1b). Thereby strongly different gold–gold distances are found. The shortest bond lengths are those of Au2‐Au4 and Au3‐Au4 with a length of 259.0 and 259.5 pm respectively. The bond length between Au2 and Au3 is 282.3 pm. The distances to Au1 are significantly increased with 290.4 pm (Au1‐Au3) and 298.3 pm (Au1‐Au2). Three gold atoms (Au1, Au2 and Au3) are bridged by two bidentate dppb ligands. A third dppb binds to Au4 only. Interestingly, no gold‐indium bonds are present in **1** despite the similar synthetic procedure to that of a Au_3_(dppe)_2_(InCl_2_)_3_ cluster [19].

Despite the large variety of tetrahedral [Au_4_]^2^^+^ clusters reported to date, **1** represents the first example with a distinct structural motif. In the following, we investigate this unique arrangement and its resulting characteristics: While calculations for not yet synthesized monocationic [Au_4_]^+^ clusters predict a rhombic structure [20], our computations for a [Au_4_]^2+^ cluster at the BP86/def2‐SV(P)//PBE0‐D4/def2‐TZVP level of theory (TURBOMOLE and effective core potential base sets) reveal an energetic preference of 58.6 kJ/mol for the tetrahedral over the rhombic structure (calculations without any ligands). This energetic preference can be attributed to the distribution of cluster electrons within the cluster, which can only be evenly distributed over all gold atoms in the tetrahedron. To investigate the ligand influence, we calculated the minimum structure for a hypothetical [Au_4_(PPh_3_)_4_]^2+^ cluster with monodentate PPh_3_ ligands in lieu of the bidentate dppb. The only minimum structure found is the tetrahedron. Since **1** is planar, the bidentate ligands seem to be the reason for the unique structure and the cluster must therefore be considered from a different perspective. The gold–gold distances in **1** differ significantly (259.0/259.5 pm vs 290.4/298.3 pm). Partitioning the cluster along the longer gold–gold distances yields an Au_3_ triangle consisting of Au2, Au3, and Au4 and the single gold atom Au1. The triangle is analogous to the [Au_3_]^+^ cluster, exhibiting gold–gold distances of 265.4 pm on average [21]. This is nearly equivalent to the average bond length of the corresponding triangle in **1** (267 pm). The distances between this triangle and the gold atom Au1 exceed these lengths by 30 pm. A comparable distance is observed between the two Au(I) atoms in the reagent dppb(AuCl)_2_ at 299.6 pm [22]. Therefore, our proposed model of **1** is that of two subunits, the [Au_3_]^+^ triangle and the single gold atom [Au1]^+^_,_ connected by bidentate phosphine ligands and aurophilic interactions.

The following DFT calculations of **1**, based on the BP86/def2‐SV(P)//PBE0‐D4/def2‐TZVP level of theory (TURBOMOLE and effective core potential base sets), further substantiate our model. The theoretical optimized gold–gold distances exhibit similar values to the experimental ones (Table S1). The bond lengths between Au1 and Au2 or Au3 are 296.5 pm, which falls within the range of the experimental bond lengths. Notably, the distorted experimental structure becomes a deltoid structure. Therefore, we assume, that the distortion between Au1 and Au2 or Au3 can be attributed to packing effects caused by the [InCl_4_]^−^ anions in the crystal, whereas the pronounced differences in distances, such as between Au1‐Au2 and Au4‐Au2, are of electronic origin. A similar effect is observed for the tetrahedral [Au_4_(PPh_3_)_4_]I_2_ cluster, in which the Au–Au distances bridged by iodine atoms are elongated by approximately 15 pm [9].

The HOMO‐1 extends over the Au_3_ triangle in a highly symmetric way (Figure 2b). The shared electron number (SEN) between these three gold atoms is 0.52 for the bonds between Au4 and Au2 or Au3 and 0.53 for the bond between Au2 and Au3 [23]. Therefore, the [Au_3_]^+^ triangle may be described as a 2e σ‐aromatic system (NICS = −24.52 ppm) [24, 25]. In contrast, there are no occupied orbitals between Au1 and Au2 or Au3 and the bonding is neglectable with a SEN of 0.06. The calculated atomic charge of Au1 is 0.262, indicating that Au1 is in contrast to the other three gold atoms positively charged (Au4: −0.077, Au2: −0.378, Au3: −0.377). The high electron density at those gold atoms is caused by the phosphine ligands, with positive charges on the phosphorus atoms ranging from 0.619 to 0.946. The HOMO and the HOMO‐2 are dominated by d‐orbitals of the gold atoms (Figure 2a,c). Conversely, the LUMO and higher unoccupied orbitals are mainly located on the ligands (Figure 2d–f). The calculations, thus support the proposed structural model of **1** in form of two subunits: The positively charged and electronically isolated Au1 and the remaining three gold atoms in form of a 2e σ‐aromat. No gold‐gold bonds have been calculated between these subunits (SEN = 0.06) and their proximity is caused by the bidentate ligands.

**FIGURE 2:**
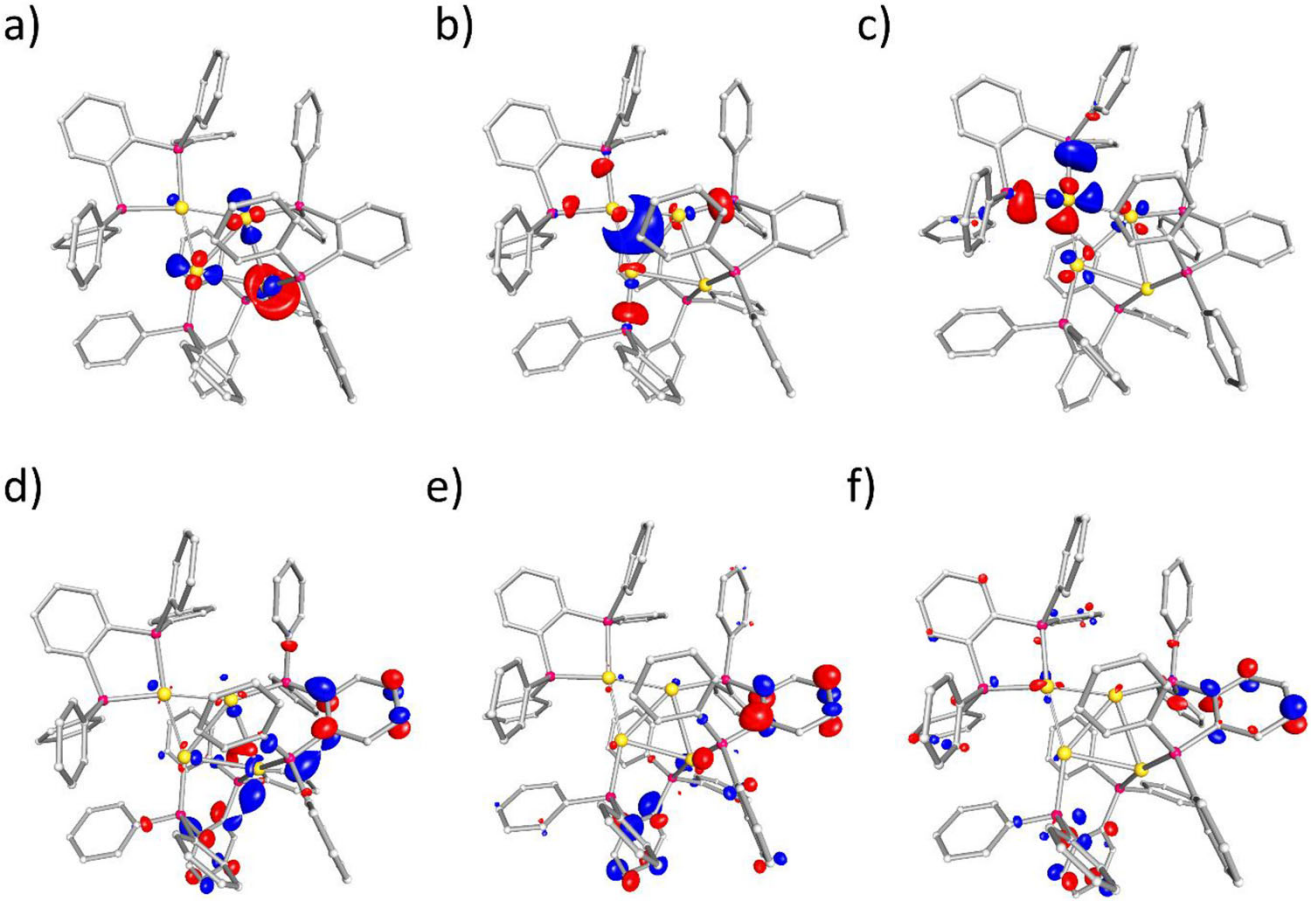
The molecular orbitals (a) HOMO‐2, (b) HOMO‐1, (c) HOMO, (d) LUMO, (e) LUMO+1 and (f) LUMO+2, calculated on the BP86/def2‐SV(P)//PBE0‐D4/def2‐TZVP level of theory, starting from the experimental structure of **1** in the solid state (iso = 0.05).

Compound **1** exhibits luminescence in both acetonitrile solution (Figure S7) and the solid state (Figure 3). A polycrystalline sample of **1** emits at 770 nm upon excitation at 375 nm at room temperature (Figure 3e). The quantum yield at this excitation wavelength was measured as 17.1% (n = 3, SD = 3.6%). Such a luminescence is similar to that of other small gold clusters, particularly a [Au_8_(dppp)_4_Cl_2_](PF_6_)_2_ cluster, which emits at 710 nm in the solid state, a [Au_25_(SR)_18_]^−^ cluster with an emission at 700 nm as well as an [Au_13_(dppe)_5_Cl_2_](PF_6_)_3_ cluster at 800 nm [26, 27, 28].

**FIGURE 3:**
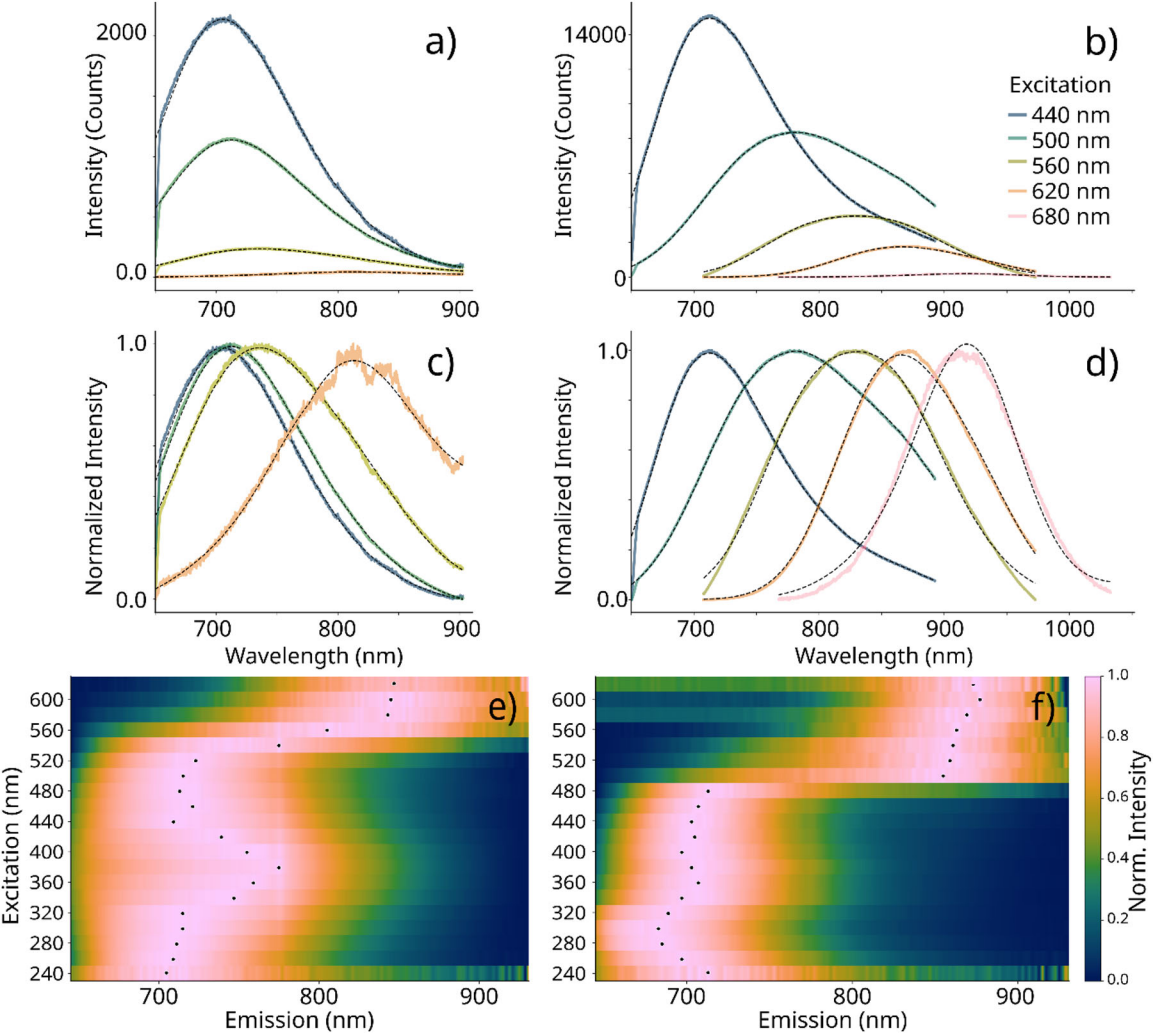
Spectral response to excitation wavelength in a polycrystalline sample of **1**. (a) and (c) Emission spectra collected under focused laser excitation with different excitation wavelengths at room temperature. Dashed lines are guides to the eye. (b) and (d) Emission spectra collected under focused laser excitation with different excitation wavelengths at 77 K. (c) and (d) show the min–max normalized spectra. (e) and (f) show the normalized emission spectra heat maps of the polycrystalline sample under broadband illumination at room temperature (e) and 77 K (f), the maxima are marked via black dots as a guide to the eye [29].

Excitation of the polycrystalline sample at 620 nm at room temperature results in a bathochromic shift of the emission maximum to 812 nm (Figure 3a,c; orange). We investigated this unusual excitation‐dependent emission in further detail in two independent measurements, first under tunable laser illumination (FWHM ∼1 nm) from 440 to 680 nm in 10 nm steps (Figure 3a–d) and second under broadband (FWHM ∼6 nm) illumination from 240 to 620 nm in 20 nm steps (Figure 3e,f), both at room temperature and 77 K.

Both measurement series resulted in qualitatively similar results and small differences can be explained by the usage of two different experimental measurement systems. At room temperature (Figure 3a,c,e), excitation at 440 nm under tunable laser illumination produces an emission at 705 nm. Upon excitation at 500 nm, the emission shifts to 713 nm. A more pronounced bathochromic shift is observed after excitation at 620 nm where the emission appears at 812 nm. Excitation at higher wavelengths wasn't feasible due to the decreasing emission intensity. A change in the quantum yield over the emission range can be estimated based on the emission intensity, with its maximum occurring in the range between 350 and 400 nm and decreasing toward higher wavelengths.

At 77 K (Figure 3b,d,f), excitation of **1** at 440 nm yields an emission maximum at 713 nm. With increasing excitation wavelengths, the emission exhibits a more pronounced bathochromic shift compared to room temperature giving rise to maxima at 782 nm (500 nm excitation), 831 nm (560 nm excitation), and 866 nm (620 nm excitation). Due to an enhanced emission intensity at 77 K, it was possible to observe an emission maximum at 918 nm upon excitation at 680 nm. The excitation wavelengths below 440 nm are examined under broadband illumination (Figure 3e,f). Excitation at 240 nm produces an emission maximum at 705 nm. As the excitation wavelength increases, the emission maximum shifts to longer wavelengths, reaching approximately 760 nm at 360 nm excitation. Upon further increase of the excitation wavelength, the emission undergoes a hypsochromic shift (Figure 3e). The excitation of **1** at 77 K at 240 nm produces an emission maximum at 705 nm. Between 240 and 440 nm, only a reduced bathochromic shift was observed (Figure 3f). Excitation at 500 nm resulted in a very broad emission spectrum featuring two maxima at 750 and 860 nm, indicating the presence of at least two emissive states, which will be discussed in more detail later. Overall, the spectra recorded using the tunable laser and broadband illumination are comparable, with the latter producing broader emission profiles. This can be assigned to a broader excitation and a different spectral excitation energy density. A similar shift is observed in solution; however, the luminescence is less intense, most likely due to additional relaxation pathways.

The broad emission spectrum observed under excitation at 500 nm suggests the presence of multiple distinct emission bands. To determine the number and nature of these emissive states, we measured the lifetimes of **1** following excitation at 520 nm at emission wavelengths between 650 and 890 nm in 30 nm intervals. For clarity, only the lifetimes measured at 650 and 890 nm are discussed here.

Room‐temperature measurements following excitation at 520 nm and monitored at 650 nm reveal three distinct lifetimes: a short‐lived excited state in the sub‐nanosecond range (0.54 ns) and two longer‐lived states at 1.9 and 4.5 µs (Table S8). The 4.5 µs lifetime dominates, accounting for 88% of the microsecond‐regime emission. In contrast, when monitored at 890 nm, the relative contribution of the nanosecond lifetime decreases to 30%, and the 1.9 µs component becomes prominent. At 77 K, a short‐lived excited state is observed, detected both at 650 and 890 nm, with a lifetime of 0.68 ns. Two excited states with longer lifetimes are present (3.4 µs and 11.3 µs). When measured at 650 nm, their relative intensities are 12% and 29%, respectively; the contribution of the longer lifetime rises to 69% when measured at 860 nm. In addition to these three lifetimes, two further lifetimes (7.3 ns and 24.1 µs) are observed. The different lifetimes measured at room temperature and at 77 K indicate that the emission spectrum arises from multiple excited states. These states are temperature‐dependent, leading to a corresponding temperature dependence of the emission spectra.

Since the emission spectra also depend on the excitation wavelength, lifetimes were measured following excitation at 375 nm. At room temperature, three distinct lifetimes were observed at 590 nm. Additional to a lifetime at 4.7 ns, two lifetimes in the microsecond‐range are 1.7 µs (27%), similar to those under 520 nm excitation, and 4.3 µs (73%). The lifetimes and their relative intensities remain similar when measured at 890 nm. At 77 K, comparable lifetimes are 2.3 ns, 2.7 µs (27%) and 6.2 µs (71%). They retain similar relative intensities when measured at 890 nm (19% and 74%). In addition to the room‐temperature measurements, a component with a lifetime of 22.8 µs is observed.

To investigate the lifetime evolution under excitation wavelength tuning we collected lifetime data systematically under pulsed laser excitation (150 kHz, FWHM ∼1 nm) between 440 and 630 nm in 10 nm steps. This leads to a long‐lived component that strongly depends on the excitation wavelength, varying between 3 and 7 µs, while its relative contribution decreases with increasing excitation wavelength compared to a shorter, nanosecond‐scale lifetime (Figure S15). In accordance with the temperature and excitation wavelength dependence of the luminescence lifetime and the emission spectra also the excitation spectra are affected by these parameters (Figure S7).

Excitation‐dependent emission is a rare, yet highly sought‐after property of a material, with potential applications in the fields of bioimaging, sensing and even anti‐counterfeiting [30, 31, 32]. A common strategy employed in the design of such materials involves the introduction of two or more luminescent species within a host material, such as a polymer [33, 34].

Excitation‐dependent properties have been reported for powdery samples of a platinum d^8^‐ and a gold d^10^‐complex [16, 17]. It is noteworthy that these excitation‐dependent properties are not observed in a highly ordered crystalline state, indicating that they depend on different intermolecular platinum–platinum and gold–gold distances, respectively. This explanation has also been used to describe a recently published polydisperse sample of gold clusters that exhibit similar properties [35]. Additionally, this phenomenon has so far been observed only for an Au_20_ cluster, and only in solution [18].

To exclude the possibility that the observed optical characteristics of **1** arise from structural or chemical impurities, measurements were performed on single crystals of **1** (Figure 4a) using a custom‐built cryogenic confocal setup.

**FIGURE 4:**
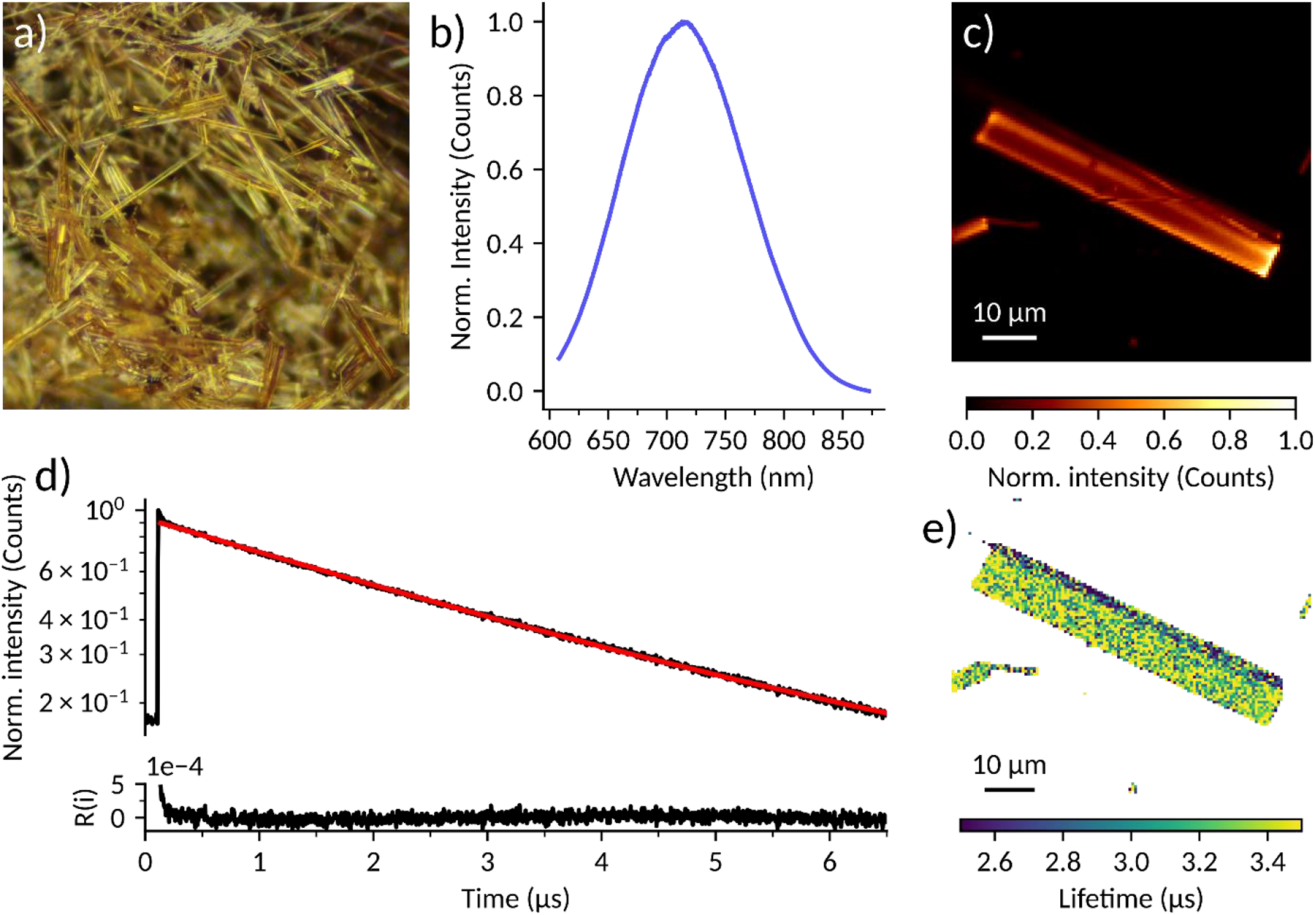
(a) As prepared crystalline sample of **1** from which the single crystals are picked and (b) the normalized single crystal PL spectrum measured with excitation at 488 nm under air. (c) Single crystal confocal photoluminescence intensity scan under 450 nm excitation and (d) averaged photoluminescence decay histogram (black), collected under 150 kHz pulsed excitation with monoexponential fit (red). The residuals of the fit are shown in black. (e) shows the lifetime map of the crystal.

The excitation of single crystals of **1** at 488 nm at room temperature results in the emission of a bright red luminescence with an emission maximum at 725 nm (Figure 4b). Figure 4c shows a luminescence scan image of a single crystal of **1**, excited at 450 nm. The average lifetime histogram fitted monoexponentially is presented in Figure 4d. The average lifetime was determined with 3.258 µs under 488 nm excitation, almost uniform over the entire single crystal (Figure 4e). These results are generally similar to the emission band and lifetimes of the polycrystalline sample.

In order to conduct a more thorough investigation of this phenomenon and to exclude that the found phenomenon is caused by chemical impurities as in the case of Au(I) complexes in literature, the emission of single crystals of **1** was measured at 77 K under nitrogen atmosphere. The results are displayed in Figure 5 and Figure S10. A wavelength range between 410 and 650 nm was used for excitation, with measurements taken at 10 nm intervals. Additional excitations were performed at 488, 532, and 633 nm corresponding to typical laser wavelengths. The excitation spectra of three measured single crystals are shown in Figure 5a. The normalized emission spectra depending on the excitation wavelength are presented as a heatmap in Figure 5b and selected emission spectra are shown in Figure 5c. Excitation at 440 nm results in an emission at 719 nm. The emission wavelength increases with excitation wavelength and **1** emits at 779 nm excited at 560 nm and at 870 nm for an excitation at 680 nm. The excitation‐dependent emission exhibits a linear relationship within the range of 410 to 560 nm, with a shift response of approximately 0.3 nm per nm of excitation (nm_em_/nm_ex_). This shift response increases almost tenfold for excitation between 560 and 590 nm and decreases again at higher wavelengths. Additional measurements at short excitation wavelengths following a full wavelength sweep confirm that the shift is not due to a second species generated by illumination (Figure S13), but is fully reversible. In addition to the emission wavelength, the intensity and the luminescence lifetimes (Figure 5d; Table S10) undergo corresponding changes.

**FIGURE 5:**
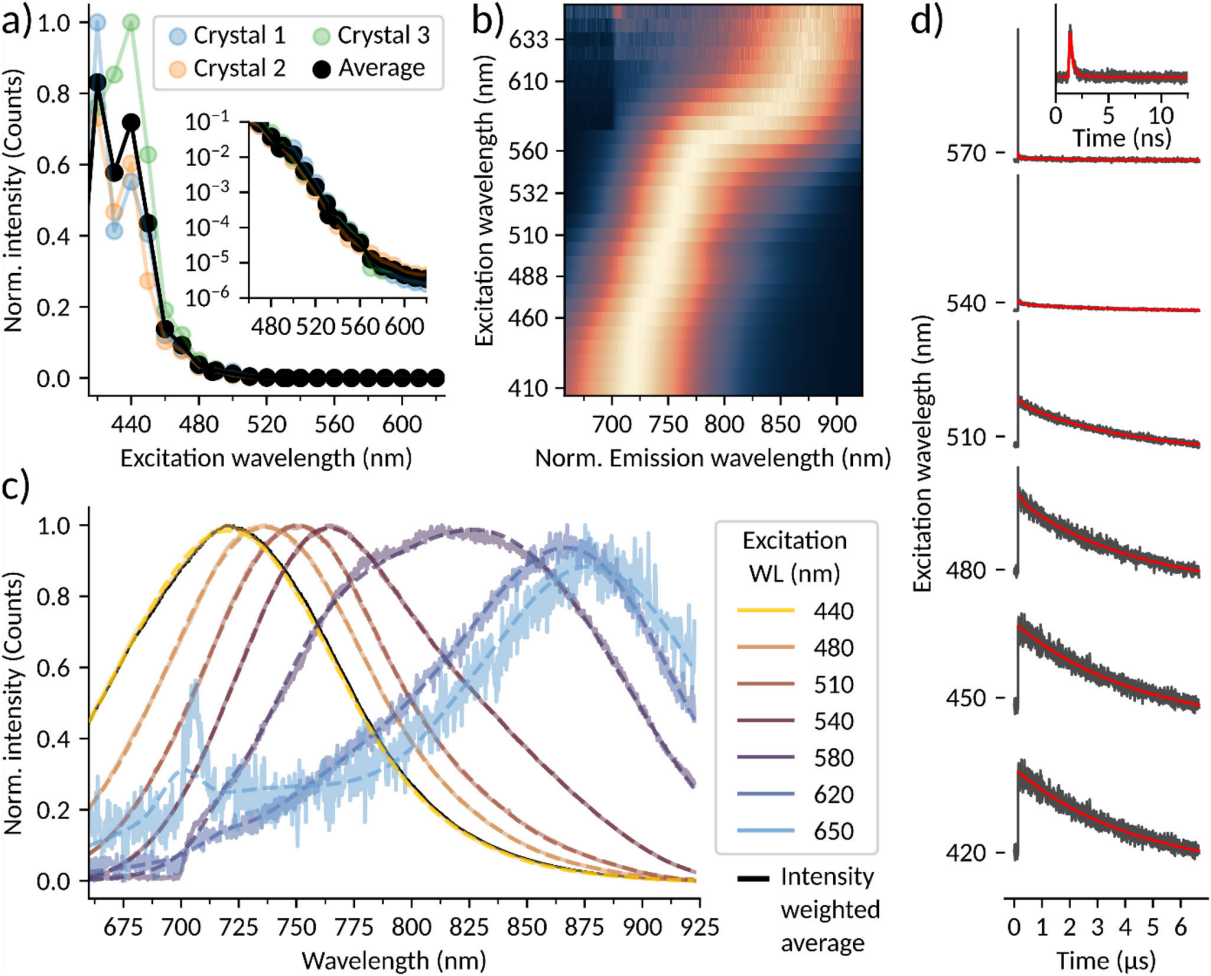
Spectroscopic characterization of **1** at 77 K in nitrogen atmosphere: (a) Excitation spectra collected from three individual single crystals. The black data points represent the average excitation spectrum of all three measured crystals. (b) Emission spectrum plotted normalized line wise, as a heat map. (c) Spectral shift plotted normalized under excitation wavelength tuning. The sum over all excitation spectra normalized to the excitation power is shown in black. (d) Normalized emission decay histograms (black) collected at different excitation wavelengths under 150 kHz pulsed laser excitation and biexponential fit (red). Additionally, a biexponentially fitted emission decay histogram collected under 80 MHz pulsed laser excitation at 570 nm is presented to better visualize the fast luminescence decay contribution dominating the histogram at lower excitation energies [29].

For excitations at 77 K in the blue spectral region, the average lifetime is dominated by a long contribution at 4 to 5 µs (Figure S15, Table S10). However, with an increase in the excitation wavelength, a very fast emission with lifetimes of several hundred picoseconds becomes prominent and dominates at 540 nm and larger wavelengths excitation (Figure 5d). Additionally, an intermediate lifetime contribution is found (in average 140 ns). All measurements were conducted at a total of three single crystals. While the overall shift response is similar for all measured crystals, the excitation ranges with linear shift response vary and seem to depend on different factors like crystal size and orientation (Figure S10). The emission spectra collected from single crystals are similar to those of the polycrystalline sample, which is an ensemble of single crystals. Therefore, chemical and structural impurities can be ruled out as the origin of the observed optical behavior. The excitation‐dependent emission of **1** in the crystalline state confirms that all clusters share a similar structural ground state and enables, for the first time, a detailed study in highly ordered chemical and structural pure sample.

Several distinct emission bands, although rare, are known for several gold clusters. The [Au_25_(SG)_18_]^−^ cluster (SG = glutathionate) for example exhibits emission at 700 and 1000 nm. The proposed mechanism involves relaxation from core states to both core and surface states, from which the respective emissions arise [27]. The Au_42_(PET)_32_ cluster (PET = 2‐phenylethanethiolate) displays emission at 806 nm from a first excited singlet state and at 1040 nm from an excited triplet state, between which intersystem crossing occurs [36]. More recently, a [Au_20_(CZ‐PrA)_16_]^2^^−^ cluster (CZ‐PrAH = 9‐(prop‐2‐yn‐1‐yl)carbazolate) was reported to show dual emission from two triplet charge‐transfer states, including ligand‐to‐kernel and kernel‐based transitions, at 820 and 940 nm upon excitation at 340 nm. For this cluster, the emission was shown to depend on the excitation wavelength too [18].

The emission spectra of **1**, collected under all excitation wavelengths are composed of multiple emission bands (Figure 5c). The sum of all emission spectra normalized to the laser intensity (Figure 5c, black line) closely resembles the emission spectrum under short‐wavelength excitation (440 nm, Figure 5c, blue line) due to the highest excitation efficiency at short wavelengths. Increasing the excitation wavelength leads to a selective emission from lower energetic states. This results in the observed red‐shift with increasing excitation wavelengths. The emission spectrum response to the excitation wavelengths increases strongly when the temperature is reduced from room temperature to 77 K assumably due to reduced vibrational quenching and thermal equilibration at higher temperature. The multiple spectral bands contributing to the spectrum combined with the measured multiple luminescence decay times indicate the presence of several distinct excited states leading to the striking excitation dependent emission.

We performed geometry optimization of the ground state and TD‐DFT calculations (Orca, all electron calculations with scalar relativistic Hamiltonian and spin orbit coupling, see SI for more details) to obtain information about the nature of the transitions in **1**. The calculated absorbance spectrum with the underlying transitions from the geometry optimized singlet ground state is shown as well as the steady‐state absorbance spectrum in acetonitrile (Figure S8). The predicted spectrum and the experimental spectrum are in reasonable agreement, besides the significant energetic shift of the predicted spectrum by about 10000 cm^−1^ to higher energies. The spin orbit coupled ground state (SOC0) is predicted to be a singlet state (more than 99%), while the energetically lowest excited state (SOC1) is predominantly a triplet sub‐state (mixture of some different non‐spin‐orbit coupled triplet states). The energetically higher (SOC2 – SOC5) excited states become more complex mixtures of different triplet states also containing contributions of energetically lower singlet excited states, however these states are dominated by the triplet character. The non‐spin‐orbit coupled singlet state S1 is formed from the HOMO‐LUMO transition, while the higher singlet states (S2‐S10) are mixtures from transition from the HOMO to the LUMO and higher unoccupied MOs. In contrast, the non‐spin‐orbit coupled triplet states (T1‐T10) are all composed of more complex mixtures from transition from the HOMO to the LUMO and higher unoccupied MOs.

Based on these TD‐DFT calculations and the depicted MOs, a possible characterization of the lowest energy excitations is a mixture of metal to ligand charge transfer (MLCT) and metal to metal charge transfer (MMCT) transitions.

Regarding the mechanism underlying the excitation‐dependent emission of **1**, we hypothesize that the observed emission originates neither exclusively from a single, fully relaxed emitter nor a classical (azulene‐type) higher‐state anti‐Kasha effect (long lifetimes at short wavelengths, but short lifetimes for long wavelengths) [37]. Beyond single‐cluster photophysics, collective effects in the crystalline state could also contribute, as intermolecular aurophilic interactions can give rise to multiple collective low‐energy emissive states that can be selectively populated by different excitation energies, a mechanism that has previously been shown to lead to excitation‐dependent emission in assembled molecules with varied Au(I)‐Au(I) distances [38].

At this point, the small size of **1** provides an advantage: positioned between Au(I) complexes and larger gold clusters, exhibiting optical properties of both, it serves as an ideal model system to study this behavior. Its tunable red to near‐infrared emission, excitation‐dependent lifetimes, high quantum yield, and remarkable solid‐state stability may provide a uniquely versatile photophysical platform. These properties could enable ratiometric sensing across multiple spectral channels and allow highly responsive optical detection under diverse experimental conditions. The excitation‐dependent emission of the cluster enables continuous tuning of the spectral overlap integral with spectrally distinct IR acceptors. This provides optical control over energy transfer selectivity in a multi‐acceptor FRET architecture, with implications for all‐optical switching and wavelength‐addressable channel selection in photonic computing. The monoclinic space group implies sought after physical effects such as second harmonic generation and parametric down conversion which in combination with excitation dependent emission will enable broad applicability of such well‐defined gold clusters in next‐generation functional materials.

## 3 Conclusion

The reaction of dppb(AuCl)_2_ with InCl results in the [Au_4_(dppb)_3_][InCl_4_][InCl_4_(THF)] cluster **1**. **1** is the first [Au_4_]^2+^ cluster that does not have the shape of a tetrahedron, but the gold atoms are arranged in a deltoid. This is attributed to the bidentate ligands, which force a planar arrangement of the gold atoms. The solid‐state emission of **1** exhibits a bright luminescence spectrum with a maximum at 770 nm, accompanied by a quantum yield (QY) of 17% under excitation at 375 nm. The luminescence of **1** depends on both temperature and excitation wavelength, producing emission bands from 705 nm after excitation at 240 nm up to 845 nm after excitation at 640 nm. At 77 K, this effect is more pronounced, with a maximum emission wavelength of 918 nm following excitation at 680 nm. Additionally, the excitation spectrum and lifetimes are also influenced by these factors, indicating the involvement of multiple excited states. Thus, **1** exhibit uniquely temperature‐ and excitation‐dependent emission in its crystalline state, combined with a novel structure in which a single gold cation is in close proximity to a delocalized electron system. This makes it a promising candidate for new metal compounds with unique optical properties.

## Author Contributions

M.S.: Synthesis, NMR studies, crystallographic section, investigations, quantum chemical calculations, original draft, visualization, review and editing. J.K.: SC luminescence measurements, original draft, visualization, review and editing. W.L.: powder luminescence measurements, quantum chemical calculations, review and editing. J.J.H.: SC luminescence measurements, review and editing. C.S.: crystallographic measurement, review and editing. C.K.: SC luminescence measurements, review and editing. M.Se.: review and editing. M.Sc.: review and editing. A. J. M.: investigations, Writing – original draft preparation, review, editing, supervision, project administration and funding acquisition. A.S.: investigations, Writing – original draft preparation, review, editing, supervision, project administration and funding acquisition. All authors have read and agreed to the published version of the manuscript.

## Conflicts of Interest

None of the authors have a conflict of interest to disclose.

## Supporting information




**Supporting File**: smll75130‐sup‐0001‐SuppMat.pdf.

## Data Availability

The data that support the findings of this study are available from the corresponding author upon reasonable request.
