# Supplementary material for: [Au4(dppb)3]2+ – A Planar Gold Cluster with Bright Excitation Wavelength Dependent Emission
Source: Small. 2026 Aug 18;22(57):e75130. doi: 10.1002/smll.75130 (PMC13659781; doi:10.1002/smll.75130)
Supplement: Supplementary file 1 [file SMLL-22-e75130-s001.pdf]

Supporting Information:

# $[Au_4(dppb)_3]^{2+}$ - A Planar Gold Cluster with Bright Excitation Wavelength Dependent Emission

Markus Strienz‡, Jakob Keck‡, Wolfgang Leis, Jonas L. Hiller, Claudio Schrenk, Christoph Kertzscher, Michael Seitz, Marcus Scheele, Alfred J. Meixner*, Andreas Schnepf*

# Table of contents

1. Synthesis ... 3
1.1. Synthesis (dppb)$(AuCl)_2$ ... 3
1.2. Synthesis $[Au_4(dppb)_3][InCl_4][InCl_4(THF)]$ ... 3
2. Structural Data ... 4
2.1. Experimental Au-Au-distances of **1** and comparison with calculated distances in a planar $[Au_4]^{2+}$-cluster ... 4
2.2. Comparison of the geometry, charge and Au-Au-distances of compounds with four gold atoms 4
2.3 Comparison of the gold-chlorine distances ... 5
3. Calculations ... 5
3.1 Calculations with TURBOMOLE ... 5
3.2 Calculations with Orca ... 14
4. Optical measurements ... 14
4.1. Luminescence measurements in solution and powder samples (broadband excitation) ... 14
4.2. Luminescence under focused laser excitation ... 17
4.2.1 Single crystalline sample ... 17
4.2.2 Powder sample ... 21
4.3. Quantum Yield ... 22
5. Crystallography ... 23
6. Mechanistic discussion ... 25
7. References ... 26

# 1. Synthesis

All reactions are performed under inert conditions using an argon atmosphere. THF and $Et_2O$ are dried with sodium, cyclopentane with NaKa and distillated.

## 1.1. Synthesis (dppb)$(AuCl)_2$

0.321 g (1 mmol, 1 eq.) (THT)AuCl are suspended with 0.223 g (0.5 mmol, 0.5 eq.) dppb in THF. The solvent is removed after one hour under reduced pressure and gives a crude white product.

Yield: 0.441 g (97 %);

$^{1}$H-NMR ($CDCl_3$, 300 MHz): δ[ppm] = 7.1-7.6 (m, 24H);

$^{31}$P-NMR ($CDCl_3$, 121,5 MHz): δ[ppm] = 24.7 (s, 2P);

## 1.2. Synthesis $[Au_4(dppb)_3][InCl_4][InCl_4(THF)]$

0.456 g (0.5 mmol, 1eq.) (dppb)$(AuCl)_2$ are suspended with 0.075 g (0.5 mmol, 1 eq.) InCl in 20 mL $Et_2O$. The size of the InCl crystals influences the reaction time strongly. During the next three days, the suspension turns brown. 45 mL THF are added after three days, which gives a brown solution. The solution is filtrated and layered with cyclopentane. The title compound **1** crystallizes after several days in form of a yellow crystalline solid and can recrystallized with F-Benzene.

Yield: 0.35 g (54 %);

$^{1}$H-NMR (THF-$d_8$, 400 MHz): δ[ppm] = 6.62-7.93 (m, 72H);

$^{31}$P-NMR (THF-$d_8$, 162 MHz): δ[ppm] = 33.3 (s, 2P); 47.1 (s, 2P); 76.1 (s, 2P);

# 2. Structural Data

## 2.1. Experimental Au-Au-distances of **1** and comparison with calculated distances in a planar $[Au_4]^{2+}$-cluster

*Table S 1: Comparison of the Au-Au distance and representative Au-P distances (in pm) in the experimental structure with those obtained from different calculations, performed with the hybrid-functional PBE0 and Grimme's dispersion correction D4 using the effective core potential (ECP) basis set def2-TZVP in TURBOMOLE (see paragraph 3.1). In Orca (see paragraph 3.2) a comparable ECP calculation (tight geometry convergence criteria) was performed as well as a geometry optimization using the scalar relativistic exact two component (X2C) Hamiltonian with an all-electron SVP basis set.*

| | **Experimental** | **TURBOMOLE ECP (3.1)** PBE0-D4/def2-TZVP | **Orca ECP (3.2)** PBE0-D4/def2-TZVP | **Orca X2C (3.2)** BP86-D4/SVP |
|---|---|---|---|---|
| Au1-Au2 | 290.3 | 296.5 | 291.2 | 284.2 |
| Au1-Au3 | 298.3 | 296.5 | 291.1 | 284.2 |
| Au2-Au3 | 282.4 | 290.2 | 293.8 | 291,4 |
| Au2-Au4 | 259.0 | 260.5 | 261.1 | 258.4 |
| Au3-Au4 | 259.5 | 260.5 | 261.1 | 258.4 |
| Au4-P32 | 235.2 | 235.4 | 236.5 | 238.6 |
| Au1-P11 | 231.8 | 232.8 | 234.3 | 235.3 |
| Au3-P12 | 229.6 | 230.7 | 233.0 | 233.9 |

## 2.2. Comparison of the geometry, charge and Au-Au-distances of compounds with four gold atoms

*Table S 2: Comparison of the geometry, charge and Au-Au-distances of four compounds with four gold atoms.*

| | Core geometry | Core charge | Au-Au-distances [pm] (Min/Max) |
|---|---|---|---|
| $[Au_4(P^tBu_3)_4][BF_4]_2$ [1] | tetrahedral | 2+ | 270.3 / 273.0 |
| $[Au_4(PPh_3)_4]I_2$ [2] | tetrahedral | 2+ | 264.9 / 282.8 |
| $[Au_4(Ph_2PCH_2PPhCH_2PPh_2)_2(C_2Ph)_2](PF_6)_2$ [3] | planar | 4+ | 307.6 / 317.8 |
| $[Au_4(dpmp)_2(SCN)_2][SCN]Cl$ [4] | linear | 4+ | 305.7 / 315.0 |

dpmp = bis(diphenylphosphinomethyl)phenylphosphine

## 2.3 Comparison of the gold-chlorine distances

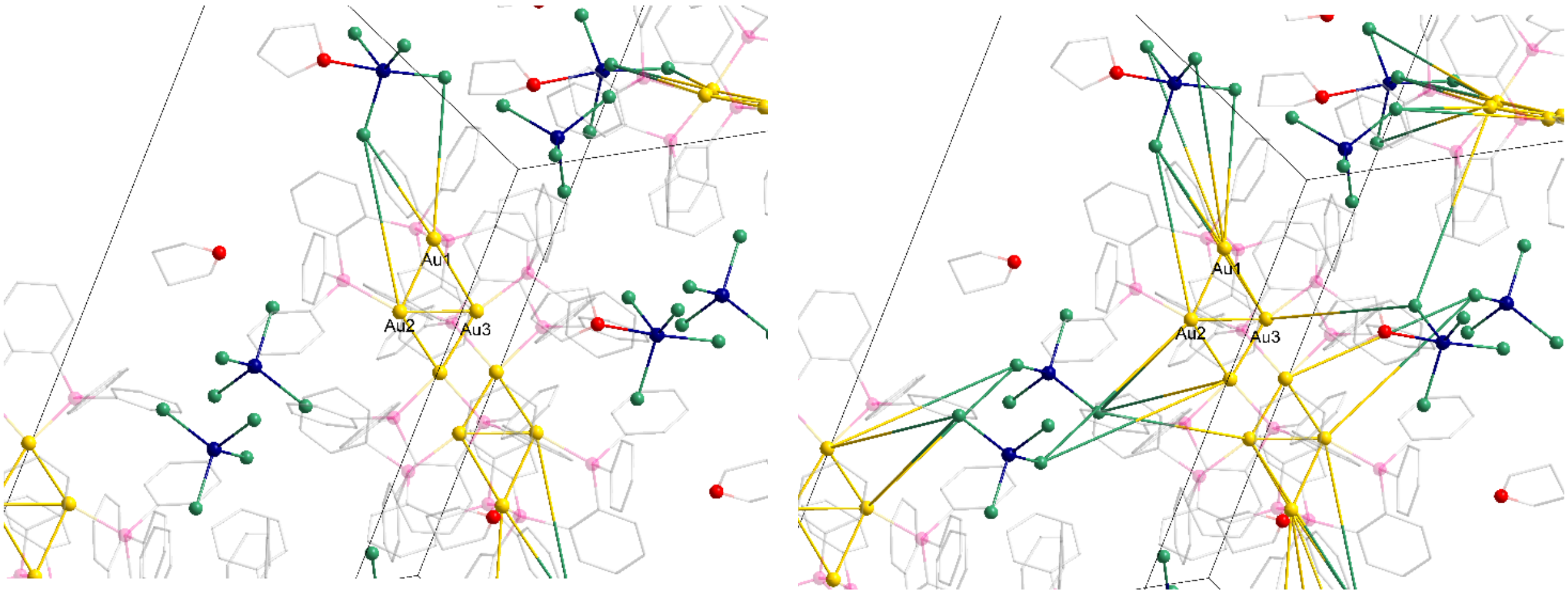


*Figure S 1: Enlarged view of the crystal structure of $[Au_4(dppb)_3][InCl_4][InCl_4(THF)]$. The gold-chlorine contacts shorter 600 pm (left) and 800 pm (right) are highlighted.*

# 3. Calculations

## 3.1 Calculations with TURBOMOLE

The following quantum mechanical calculations have been done with the program package TURBOMOLE (version 7.4.1)[5] using the graphical user interface TmoleX (version 4.4.1)[6]. The ground state geometry were preoptimized using BP86 [7, 8] or PBE0 [9] and Grimme's D4 dispersion correction [10-12] with Def2-TZVP [13] basis sets with the appropriate auxiliary basis set (Def2/J)[14] and for Au the adequate effective core potentials (Def2-ECP).[15]

$[Au_4(dppb)_3]^{2+}$:

BP86/def2-SV(P)//PBE0-D4/def2-TZVP

Total energy: --6060.65022718175 H

HOMO-LUMO: 3.595 eV

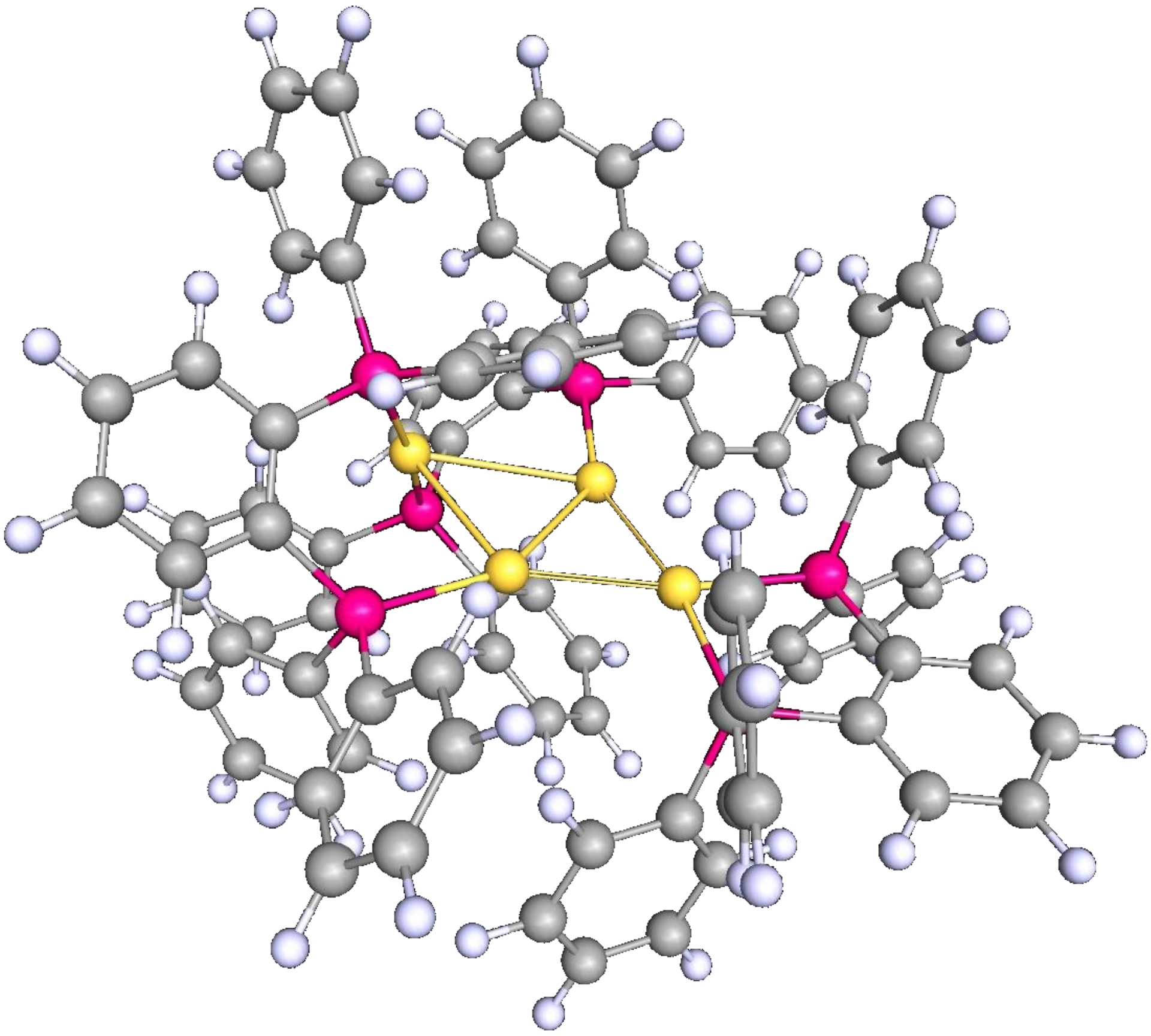

*Figure S 2: Image of the minimum structure of $[Au_4(dppb)_3]^{2+}$.*

*Table S 3: Atomic coordinates of $[Au_4(dppb)_3]^{2+}$.*

| Atom numbe r | Atom symbol | x | y | z | Atom number | Atom symbol | x | y | z |
|---|---|---|---|---|---|---|---|---|---|
| 1 | Au | 2.74402 | 25.66113 | 11.94214 | 5 | P | 2.96415 | 27.29260 | 13.58633 |
| 2 | Au | 3.84746 | 24.06194 | 14.18436 | 7 | P | 1.82696 | 24.48545 | 15.21041 |
| 3 | Au | 5.31439 | 24.20095 | 11.68745 | 8 | P | 5.50277 | 25.30378 | 9.67167 |
| 6 | Au | 6.11533 | 22.85295 | 13.76629 | 9 | P | 6.40837 | 21.62541 | 15.75222 |
| 99 | C | 10.81348 | 25.84284 | 12.81797 | 10 | P | 8.22949 | 22.07759 | 13.08396 |
| 100 | C | 10.76563 | 25.12906 | 14.00632 | 4 | P | 2.27316 | 24.23695 | 10.16352 |
| 98 | C | 10.13894 | 25.37000 | 11.69767 | 59 | C | 0.95275 | 23.01275 | 17.45857 |
| 97 | C | 10.04556 | 23.94456 | 14.08034 | 60 | C | 3.19834 | 23.71422 | 18.93891 |
| 96 | C | 9.40884 | 24.19654 | 11.76925 | 61 | C | 2.23221 | 22.88297 | 19.49137 |
| 95 | C | 7.54127 | 19.44715 | 10.09100 | 62 | C | 1.11292 | 22.53218 | 18.75073 |
| 11 | C | 0.49060 | 24.05883 | 9.93789 | 63 | C | 0.08086 | 26.26411 | 16.34597 |
| 12 | C | 2.91662 | 22.55569 | 10.31372 | 64 | C | -0.53789 | 27.47865 | 16.58985 |
| 13 | C | 2.89732 | 24.85545 | 8.56621 | 65 | C | -0.09986 | 28.61193 | 15.92693 |
| 14 | C | 4.22259 | 25.27362 | 8.37263 | 66 | C | 0.95080 | 28.52303 | 15.02782 |
| 15 | C | 6.97237 | 24.65735 | 8.83171 | 67 | C | 2.12905 | 29.19802 | 11.78022 |
| 16 | C | 5.82273 | 27.05685 | 9.96755 | 68 | C | 3.78428 | 29.95988 | 13.35759 |
| 17 | C | 0.52203 | 23.54850 | 14.38429 | 69 | C | 2.06828 | 30.46588 | 11.22868 |
| 18 | C | 1.92529 | 23.83451 | 16.89850 | 70 | C | 2.87009 | 31.48077 | 11.73547 |
| 19 | C | 1.13150 | 26.15623 | 15.43886 | 71 | C | 3.73127 | 31.22527 | 12.79247 |
| 20 | C | 1.57773 | 27.30611 | 14.77007 | 72 | C | 5.66492 | 26.92454 | 13.95391 |
| 21 | C | 2.97823 | 28.94354 | 12.85541 | 73 | C | 4.44079 | 27.41612 | 15.96992 |
| 22 | C | 4.45875 | 27.19320 | 14.59604 | 74 | C | 6.84453 | 26.87590 | 14.67578 |
| 23 | C | 5.17818 | 20.31020 | 15.82993 | 75 | C | 6.82383 | 27.09397 | 16.04620 |
| 24 | C | 6.47197 | 22.39515 | 17.38338 | 76 | C | 5.62372 | 27.36101 | 16.69145 |
| 25 | C | 8.03253 | 20.80526 | 15.58319 | 77 | C | 5.39260 | 19.08808 | 15.19961 |

| | | | | | | | | | |
|---|---|---|---|---|---|---|---|---|---|
| 26 | C | 8.83429 | 21.03703 | 14.45891 | 78 | C | 3.92477 | 20.59747 | 16.37313 |
| 27 | C | 8.47839 | 21.05656 | 11.61679 | 79 | C | 4.36279 | 18.16131 | 15.11287 |
| 28 | C | 9.36391 | 23.47317 | 12.96235 | 80 | C | 3.12001 | 18.44684 | 15.65872 |
| 29 | C | -0.08271 | 22.84821 | 9.56290 | 81 | C | 2.90563 | 19.66445 | 16.29515 |
| 30 | C | -0.31425 | 25.18222 | 10.11719 | 82 | C | 6.82481 | 23.74055 | 17.44431 |
| 31 | C | -1.45208 | 22.76728 | 9.35892 | 83 | C | 6.25404 | 21.68301 | 18.56074 |
| 32 | C | -2.24797 | 23.89214 | 9.51849 | 84 | C | 6.98974 | 24.36166 | 18.67224 |
| 33 | C | -1.67837 | 25.10089 | 9.89830 | 85 | C | 6.79473 | 23.64313 | 19.84384 |
| 34 | C | 2.79699 | 21.91775 | 11.54584 | 86 | C | 6.41755 | 22.30749 | 19.78666 |
| 35 | C | 3.48255 | 21.87987 | 9.23644 | 87 | C | 8.49910 | 19.96826 | 16.59526 |
| 36 | C | 3.24111 | 20.61677 | 11.70443 | 88 | C | 9.74407 | 19.37301 | 16.50171 |
| 37 | C | 3.80967 | 19.94639 | 10.63045 | 89 | C | 10.54463 | 19.61274 | 15.39196 |
| 38 | C | 3.93165 | 20.57812 | 9.40001 | 90 | C | 10.08954 | 20.43669 | 14.37853 |
| 39 | C | 2.01349 | 24.89340 | 7.49024 | 91 | C | 9.71709 | 20.92391 | 10.99320 |
| 40 | C | 2.42175 | 25.33651 | 6.24235 | 92 | C | 7.38817 | 20.32656 | 11.15117 |
| 41 | C | 3.72996 | 25.74529 | 6.04984 | 93 | C | 9.86631 | 20.04550 | 9.93221 |
| 42 | C | 4.61934 | 25.70726 | 7.11053 | 94 | C | 8.78234 | 19.29979 | 9.48680 |
| 43 | C | 7.10206 | 23.27053 | 8.76237 | 50 | C | 5.21640 | 29.38138 | 9.86265 |
| 44 | C | 7.95894 | 25.47051 | 8.28380 | 51 | C | 6.32353 | 29.71044 | 10.62797 |
| 45 | C | 8.20102 | 22.70523 | 8.14050 | 52 | C | 7.18443 | 28.71245 | 11.06798 |
| 46 | C | 9.19143 | 23.51931 | 7.60570 | 53 | C | 0.67378 | 22.16371 | 14.29599 |
| 47 | C | 9.07020 | 24.89923 | 7.67959 | 54 | C | -0.55961 | 24.16191 | 13.76274 |
| 48 | C | 4.96333 | 28.05828 | 9.53048 | 55 | C | -0.25488 | 21.40359 | 13.60845 |
| 49 | C | 6.93186 | 27.39125 | 10.74632 | 56 | C | -1.33520 | 22.02063 | 12.98968 |
| 58 | C | 3.05290 | 24.17819 | 17.64367 | 57 | C | -1.48402 | 23.39599 | 13.06723 |
| 134 | H | -2.29938 | 25.97830 | 10.03505 | 140 | H | -2.32052 | 23.88065 | 12.57988 |
| 171 | H | 3.15160 | 20.12971 | 12.66899 | 141 | H | 10.83125 | 19.94484 | 9.44917 |
| 170 | H | 1.52580 | 21.68045 | 14.76310 | 142 | H | 10.09245 | 18.72326 | 17.29583 |
| 169 | H | 7.88599 | 19.78014 | 17.46843 | 143 | H | -0.56862 | 29.57151 | 16.10907 |
| 168 | H | 4.37268 | 20.05514 | 8.55964 | 144 | H | 6.69208 | 18.87832 | 9.73122 |
| 167 | H | 6.92197 | 24.12888 | 20.80445 | 145 | H | 4.36443 | 32.01353 | 13.18243 |
| 101 | H | 9.84167 | 25.53742 | 7.26459 | 146 | H | 11.52353 | 19.15434 | 15.31598 |
| 102 | H | 3.74559 | 21.55304 | 16.85376 | 147 | H | 5.94978 | 20.64332 | 18.52524 |
| 103 | H | -1.89647 | 21.82146 | 9.07214 | 148 | H | 1.40330 | 30.65973 | 10.39535 |
| 104 | H | 4.53565 | 17.21250 | 14.61832 | 149 | H | -3.31767 | 23.82570 | 9.35661 |
| 105 | H | 4.45361 | 29.76879 | 14.18759 | 150 | H | 3.57552 | 22.36234 | 8.27084 |
| 106 | H | 11.29206 | 25.49203 | 14.88144 | 151 | H | 2.83002 | 32.47137 | 11.29731 |
| 107 | H | 5.60618 | 27.53398 | 17.76104 | 152 | H | 2.35509 | 22.50437 | 20.49955 |
| 108 | H | 4.09405 | 27.81320 | 8.93265 | 153 | H | 6.24695 | 21.75071 | 20.70070 |
| 109 | H | 5.67841 | 26.75067 | 12.88586 | 154 | H | 8.29520 | 21.62803 | 8.09227 |
| 110 | H | 0.53425 | 21.96788 | 9.42947 | 155 | H | 1.51422 | 28.40149 | 11.37428 |
| 111 | H | 6.36082 | 18.85603 | 14.77079 | 156 | H | 0.98943 | 24.57062 | 7.62287 |
| 112 | H | 1.71348 | 25.35462 | 5.42275 | 157 | H | 10.06149 | 23.07640 | 7.13487 |
| 113 | H | 7.77851 | 26.65874 | 14.16966 | 158 | H | 10.18137 | 25.92075 | 10.76479 |
| 114 | H | 8.90216 | 18.61334 | 8.65653 | 159 | H | 10.71901 | 20.61087 | 13.51416 |
| 115 | H | 4.15763 | 18.92662 | 10.75117 | 160 | H | 10.01442 | 23.39289 | 15.01302 |
| 116 | H | 1.93816 | 19.88737 | 16.73070 | 161 | H | 4.07720 | 23.98417 | 19.51017 |
| 117 | H | 6.51559 | 30.74467 | 10.88773 | 162 | H | 4.53960 | 30.15581 | 9.52447 |
| 118 | H | 4.06258 | 26.09234 | 5.07902 | 163 | H | 10.56578 | 21.50927 | 11.32773 |
| 119 | H | 11.37797 | 26.76633 | 12.76151 | 164 | H | 8.04920 | 28.96545 | 11.67040 |
| 120 | H | 0.35784 | 21.88269 | 19.17815 | 165 | H | 6.34734 | 22.63276 | 9.21037 |
| 121 | H | -2.05780 | 21.42673 | 12.44273 | 166 | H | -0.68592 | 25.23635 | 13.81545 |
| 122 | H | 1.28612 | 29.41914 | 14.52286 | 130 | H | 7.74656 | 27.05797 | 16.61440 |
| 123 | H | 5.64382 | 26.02166 | 6.95011 | 131 | H | 3.82572 | 24.79841 | 17.20196 |
| 124 | H | 7.87182 | 26.54913 | 8.33447 | 132 | H | 0.12850 | 26.12248 | 10.42890 |
| 125 | H | 8.86902 | 23.84308 | 10.89750 | 133 | H | 2.35712 | 22.44349 | 12.38322 |
| 126 | H | 0.07407 | 22.73533 | 16.88886 | 172 | H | 7.59665 | 26.61256 | 11.10610 |
| 127 | H | 7.26626 | 25.40833 | 18.71782 | 135 | H | 6.41638 | 20.45916 | 11.61497 |
| 128 | H | 6.95791 | 24.30227 | 16.52572 | 136 | H | -0.13277 | 20.32840 | 13.54659 |
| 129 | H | -1.35715 | 27.53463 | 17.29644 | 137 | H | 3.51013 | 27.63229 | 16.48079 |
| 139 | H | -0.26263 | 25.38281 | 16.87458 | 138 | H | 2.31783 | 17.72109 | 15.59204 |

For the NICS calculations, a dummy atom (ghost atom) was placed at the centroid of the $Au_3$ ring (5.003/23.779/13.165) in the optimized structure of $[Au_4(dppb)_3]^{2+}$. The geometry was optimized at the PBE0-D4/def2-TZVP level of theory, while the NICS value was calculated at the BP86/def2-SV(P) level using the mpshift module implemented in TURBOMOLE. The BP86/def2-SV(P) level was chosen for the NICS calculations because of the substantially higher computational cost associated with the mpshift calculations at the PBE0-D4/def2-TZVP level.

Dummy Atom: ISOTROPIC: 24.5165898 ANISOTROPIC: 40.3338475

diamagnetic part of magnetic shielding:

Trace = 18.12320259

Tensor :

| | | |
|---|---|---|
| 23.58805488 | -24.42013124 | -13.28605393 |
| -23.54604193 | -3.09394301 | -17.79515115 |
| -11.94193285 | -16.17381202 | 33.87549591 |

paramagnetic undisturbed density part of magnetic shielding:

Trace = 59.61750910

Tensor :

| | | |
|---|---|---|
| 98.36039738 | -10.63525582 | -16.88515057 |
| -6.74733722 | 37.12944554 | 11.45348748 |
| -18.64189678 | 23.43572550 | 43.36268438 |

paramagnetic disturbed density part of magnetic shielding:

Trace = -53.22412193

Tensor :

| | | |
|---|---|---|
| -94.19019304 | 22.36272059 | 22.53841619 |
| 15.47927097 | -23.20699032 | -2.24018827 |
| 19.62265280 | -19.95764331 | -42.27518243 |

total magnetic shielding:

Trace = 24.51658976

Tensor :

| | | |
|---|---|---|
| 27.75825922 | -12.69266646 | -7.63278832 |
| -14.81410818 | 10.82851221 | -8.58185194 |
| -10.96117684 | -12.69572983 | 34.96299786 |

$[Au_4(PPh_3)_4]^{2+}$:

BP86/def2-SV(P)

Total energy: -4686.34190158604 H

HOMO-LUMO: 2.948 eV

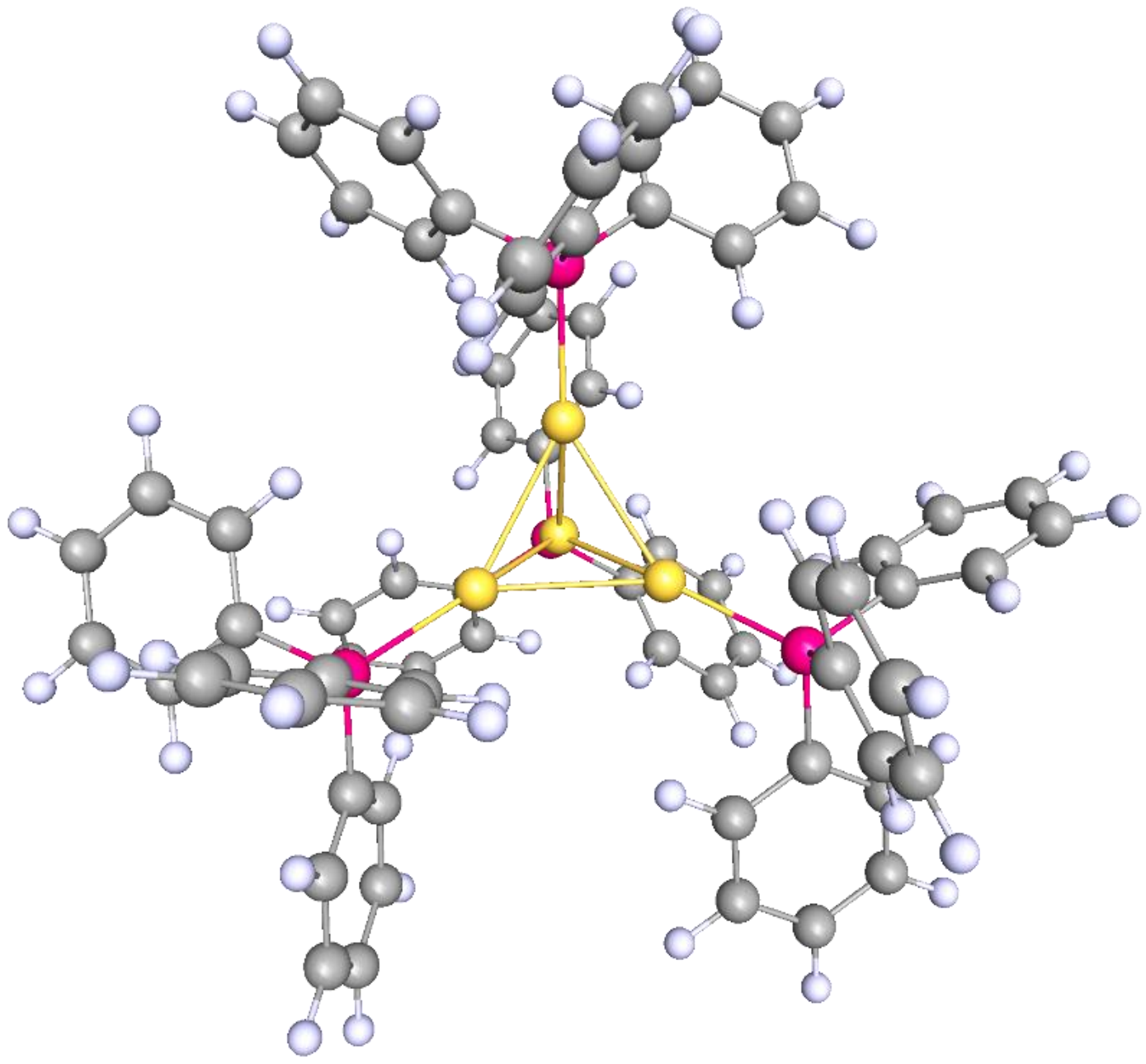

*Figure S 3: Image of the minimum structure of $[Au_4(PPh_3)_4]^{2+}$.*

*Table S 4: Atomic coordinates of $[Au_4(PPh_3)_4]^2$.*

| Atom number | Atom symbol | x | y | z | Atom number | Atom symbol | x | y | z |
|---|---|---|---|---|---|---|---|---|---|
| 1 | Au | -0.06695 | -1.25917 | 1.12521 | 86 | C | -5.87459 | 3.08656 | -2.63209 |
| 2 | Au | 0.29293 | 1.52330 | 0.83640 | 87 | C | -5.11506 | 4.26731 | -2.57058 |
| 3 | Au | -1.13948 | 0.07624 | -1.11719 | 88 | C | -3.71091 | 4.19874 | -2.50761 |
| 4 | Au | 1.65161 | -0.26083 | -0.87544 | 89 | C | -3.06658 | 2.95181 | -2.50511 |
| 5 | P | -2.94879 | 0.15264 | -2.63569 | 90 | H | -5.83988 | 0.91281 | -2.68601 |
| 6 | P | 0.44858 | 3.57543 | 1.99655 | 91 | H | -6.97460 | 3.13730 | -2.67989 |
| 7 | P | -0.39992 | -3.00976 | 2.67651 | 92 | H | -5.61973 | 5.24732 | -2.56981 |
| 8 | C | 0.63992 | 4.99959 | 0.85544 | 93 | H | -3.11293 | 5.12325 | -2.45656 |
| 9 | C | -0.00490 | 6.23101 | 1.10475 | 94 | H | -1.96513 | 2.90297 | -2.44878 |
| 10 | C | 0.19996 | 7.31622 | 0.23513 | 95 | C | -1.98670 | -2.83716 | 3.58142 |
| 11 | C | 1.04382 | 7.17972 | -0.88061 | 96 | C | -2.77931 | -3.96355 | 3.89346 |
| 12 | C | 1.68333 | 5.95223 | -1.13363 | 97 | C | -3.96665 | -3.80112 | 4.62810 |
| 13 | C | 1.48011 | 4.86216 | -0.27314 | 98 | C | -4.36700 | -2.52246 | 5.05282 |
| 14 | H | -0.66798 | 6.34628 | 1.97743 | 99 | C | -3.58127 | -1.39827 | 4.73893 |
| 15 | H | -0.30323 | 8.27636 | 0.43485 | 100 | C | -2.39695 | -1.55112 | 4.00135 |
| 16 | H | 1.20272 | 8.03423 | -1.55862 | 101 | H | -2.47242 | -4.96953 | 3.56429 |
| 17 | H | 2.34280 | 5.84144 | -2.00982 | 102 | H | -4.58107 | -4.68322 | 4.87165 |
| 18 | H | 1.97790 | 3.89880 | -0.47975 | 103 | H | -5.29807 | -2.40051 | 5.63022 |
| 19 | C | 1.88632 | 3.60806 | 3.13650 | 104 | H | -3.89469 | -0.39403 | 5.06783 |
| 20 | C | 2.68715 | 4.76254 | 3.27685 | 105 | H | -1.78751 | -0.66549 | 3.75078 |
| 21 | C | 3.75457 | 4.76633 | 4.19142 | 106 | H | -1.71917 | -3.94482 | 0.23959 |
| 22 | C | 4.02622 | 3.62624 | 4.96705 | 107 | P | 3.66187 | -0.64566 | -2.05515 |

| | | | | | | | | | |
|---|---|---|---|---|---|---|---|---|---|
| 23 | C | 3.23282 | 2.47282 | 4.82508 | 108 | C | 3.58665 | -2.17751 | -3.06243 |
| 24 | C | 2.16940 | 2.45834 | 3.90919 | 109 | C | 2.95747 | -3.32125 | -2.51972 |
| 25 | H | 2.48025 | 5.66120 | 2.67361 | 110 | C | 2.93041 | -4.52002 | -3.24924 |
| 26 | H | 4.37599 | 5.67031 | 4.29877 | 111 | C | 3.51994 | -4.58286 | -4.52527 |
| 27 | H | 4.86229 | 3.63558 | 5.68543 | 112 | C | 4.13744 | -3.44445 | -5.07152 |
| 28 | H | 3.44503 | 1.57615 | 5.42997 | 113 | C | 4.17348 | -2.24143 | -4.34535 |
| 29 | H | 1.55499 | 1.54835 | 3.79565 | 114 | H | 2.48701 | -3.27437 | -1.52221 |
| 30 | C | -1.04402 | 3.90591 | 3.01219 | 115 | H | 2.44259 | -5.41031 | -2.81992 |
| 31 | C | -2.31744 | 3.68029 | 2.44014 | 116 | H | 3.49596 | -5.52448 | -5.09790 |
| 32 | C | -3.47744 | 3.96523 | 3.17680 | 117 | H | 4.59889 | -3.49074 | -6.07146 |
| 33 | C | -3.37598 | 4.46432 | 4.48873 | 118 | H | 4.65959 | -1.35340 | -4.78081 |
| 34 | C | -2.11165 | 4.67933 | 5.06316 | 119 | C | 5.09585 | -0.82073 | -0.92339 |
| 35 | C | -0.94444 | 4.40279 | 4.32996 | 120 | C | 6.09856 | -1.78842 | -1.15140 |
| 36 | H | -2.40277 | 3.28474 | 1.41317 | 121 | C | 7.20536 | -1.86550 | -0.28831 |
| 37 | H | -4.46812 | 3.79479 | 2.72444 | 122 | C | 7.31788 | -0.98224 | 0.79909 |
| 38 | H | -4.28830 | 4.68582 | 5.06645 | 123 | C | 6.31725 | -0.02054 | 1.03136 |
| 39 | H | -2.02908 | 5.06915 | 6.09082 | 124 | C | 5.20539 | 0.05850 | 0.17842 |
| 40 | H | 0.04299 | 4.57615 | 4.78728 | 125 | H | 6.01881 | -2.48393 | -2.00242 |
| 41 | C | -4.19636 | -1.14694 | -2.28543 | 126 | H | 7.98641 | -2.62141 | -0.47113 |
| 42 | C | -4.81212 | -1.87523 | -3.32656 | 127 | H | 8.18968 | -1.04440 | 1.47086 |
| 43 | C | -5.79392 | -2.83458 | -3.02328 | 128 | H | 6.40141 | 0.67190 | 1.88483 |
| 44 | C | -6.16509 | -3.07001 | -1.68850 | 129 | H | 4.41910 | 0.80931 | 0.36954 |
| 45 | C | -5.54990 | -2.34821 | -0.64885 | 130 | C | 4.05692 | 0.72769 | -3.20682 |
| 46 | C | -4.56430 | -1.39305 | -0.94257 | 131 | C | 3.02334 | 1.25202 | -4.01710 |
| 47 | H | -4.52767 | -1.69615 | -4.37601 | 132 | C | 3.30425 | 2.27245 | -4.93867 |
| 48 | H | -6.27304 | -3.39965 | -3.83938 | 73 | C | 0.93109 | -3.06686 | 3.93860 |
| 49 | H | -6.93709 | -3.82172 | -1.45599 | 74 | C | 2.27029 | -2.88043 | 3.52522 |
| 50 | H | -5.83723 | -2.53293 | 0.39911 | 75 | C | 3.31227 | -2.96799 | 4.46144 |
| 51 | H | -4.07967 | -0.83420 | -0.12309 | 76 | C | 3.02552 | -3.22879 | 5.81415 |
| 52 | C | -2.40052 | -0.09053 | -4.37010 | 77 | C | 1.69425 | -3.40234 | 6.22985 |
| 53 | C | -2.94232 | 0.67335 | -5.42673 | 78 | C | 0.64535 | -3.32326 | 5.29743 |
| 54 | C | -2.52902 | 0.43113 | -6.74837 | 79 | H | 2.49985 | -2.66762 | 2.46667 |
| 55 | C | -1.58011 | -0.56882 | -7.02129 | 80 | H | 4.35510 | -2.82884 | 4.13267 |
| 56 | C | -1.03571 | -1.32873 | -5.96940 | 81 | H | 3.84476 | -3.29545 | 6.54871 |
| 57 | C | -1.43828 | -1.08904 | -4.64635 | 82 | H | 1.46654 | -3.60426 | 7.28921 |
| 58 | H | -3.68763 | 1.45869 | -5.22167 | 83 | H | -0.39549 | -3.46214 | 5.63165 |
| 59 | H | -2.95578 | 1.02895 | -7.57018 | 84 | C | -3.82792 | 1.76253 | -2.57736 |
| 60 | H | -1.26082 | -0.75639 | -8.05955 | 85 | C | -5.23682 | 1.83377 | -2.63640 |
| 61 | H | -0.28910 | -2.11164 | -6.18037 | 66 | C | -0.58386 | -7.16654 | 0.59909 |
| 62 | H | -1.00313 | -1.68309 | -3.82387 | 67 | C | -1.26502 | -6.06308 | 0.05229 |
| 63 | C | -0.43107 | -4.65354 | 1.86059 | 68 | C | -1.18633 | -4.80792 | 0.67506 |
| 64 | C | 0.25475 | -5.76087 | 2.40528 | 69 | H | 0.85084 | -5.64983 | 3.32537 |
| 65 | C | 0.17588 | -7.01317 | 1.77127 | 70 | H | 0.71114 | -7.87540 | 2.20129 |
| 72 | H | -1.86093 | -6.18069 | -0.86759 | 71 | H | -0.64540 | -8.15133 | 0.10762 |

$[Au_4(PPh_3)_4]^{2+}$ in Cs-symmetry:

BP86/def2-SV(P)

Total energy: -4686.30505163016 H

HOMO-LUMO: 1.639 eV

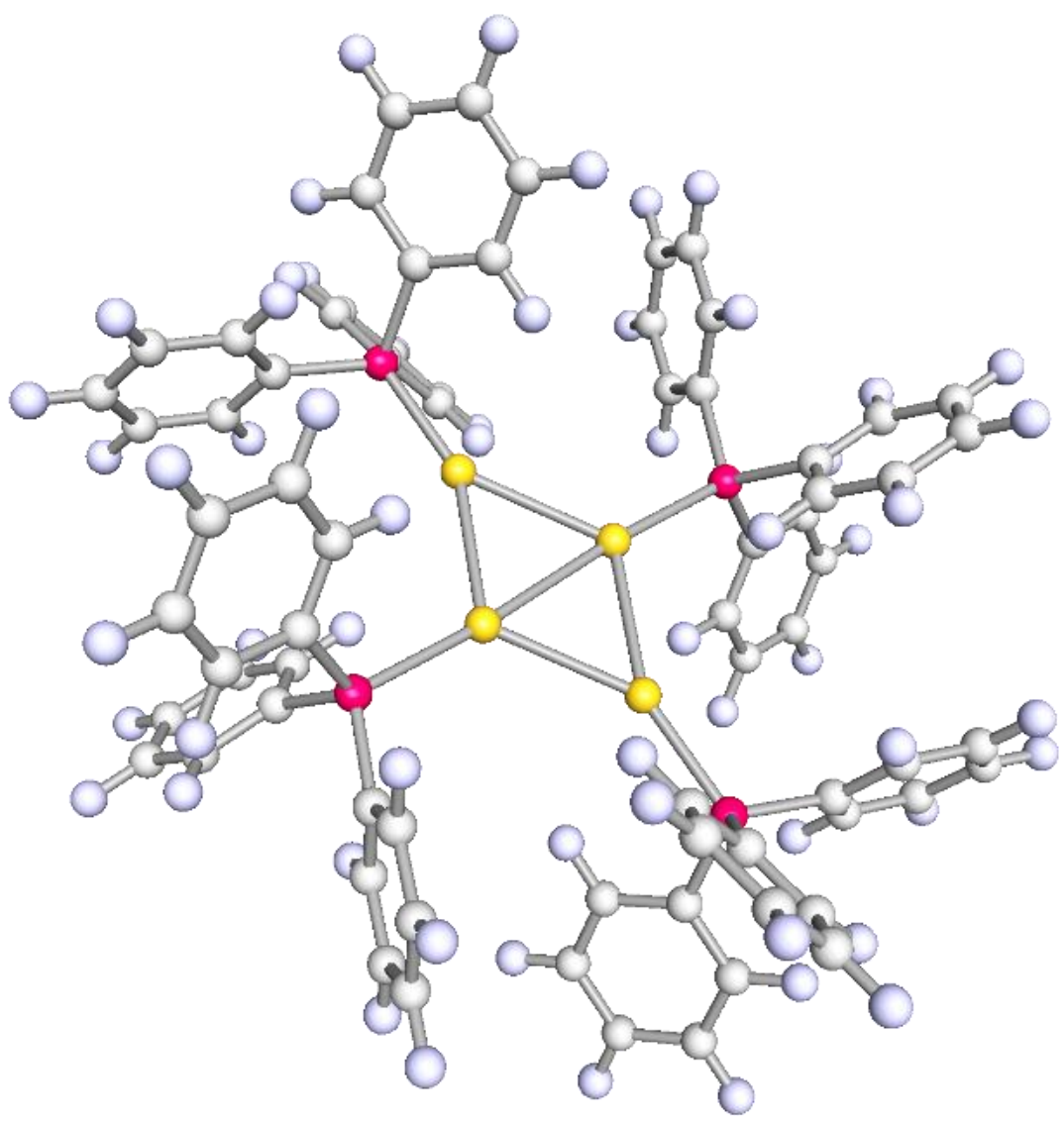

*Figure S 4: Image of the minimum structure of $[Au_4(PPh_3)_4]^{2+}$ in Cs-symmetry.*

*Table S 5: Atomic coordinates of $[Au_4(PPh_3)_4]^{2}$ in Cs-symmetry.*

| Atom number | Atom symb ol | x | y | z | Atom number | Atom symbol | x | y | z |
|---|---|---|---|---|---|---|---|---|---|
| 1 | Au | -0.18641 | 2.38989 | 0.00000 | 73 | C | -0.52858 | -6.76413 | -1.81960 |
| 2 | Au | 0.18642 | -2.38977 | 0.00000 | 84 | C | -5.88602 | 0.77278 | -1.80317 |
| 3 | Au | 1.39788 | 0.08173 | 0.00000 | 83 | C | -6.37049 | 1.43382 | -2.94233 |
| 4 | Au | -1.39790 | -0.08160 | 0.00000 | 82 | C | -5.48635 | 2.13094 | -3.78622 |
| 5 | C | 2.06950 | 5.05809 | -3.44411 | 81 | C | -4.11436 | 2.16660 | -3.48750 |
| 6 | C | 1.96063 | 6.42244 | -3.76063 | 80 | C | -3.62510 | 1.51046 | -2.34476 |
| 7 | C | 1.19092 | 7.27367 | -2.94686 | 79 | C | -4.50636 | 0.80927 | -1.49318 |
| 8 | C | 0.52854 | 6.76426 | -1.81961 | 139 | C | 1.41440 | 4.54182 | -2.31333 |
| 9 | C | 4.59508 | 1.71163 | 0.00000 | 77 | H | -1.09973 | -8.34361 | -3.19447 |
| 10 | C | 4.85354 | 2.37638 | -1.22076 | 76 | H | -2.47278 | -6.82618 | -4.64925 |
| 11 | C | 5.38221 | 3.67768 | -1.21614 | 75 | H | -2.66435 | -4.38673 | -4.08466 |
| 12 | C | 5.64981 | 4.32995 | 0.00000 | 74 | H | -1.50223 | -3.47009 | -2.06521 |
| 13 | C | 5.38221 | 3.67768 | 1.21614 | 85 | H | -2.54542 | 1.53791 | -2.11469 |
| 14 | C | 4.85354 | 2.37638 | 1.22076 | 86 | H | -3.41627 | 2.70178 | -4.15179 |
| 15 | C | 0.63920 | 5.39132 | -1.49522 | 87 | H | -5.87055 | 2.64151 | -4.68455 |
| 16 | C | 1.41440 | 4.54182 | 2.31333 | 88 | H | -7.44692 | 1.39815 | -3.17670 |
| 17 | C | 2.06950 | 5.05809 | 3.44411 | 89 | H | -6.58987 | 0.21355 | -1.16609 |
| 18 | C | 1.96063 | 6.42244 | 3.76063 | 67 | H | 0.08506 | -7.44099 | 1.20359 |
| 19 | C | 1.19092 | 7.27367 | 2.94686 | 66 | H | -1.09973 | -8.34361 | 3.19447 |
| 20 | C | -1.89621 | 5.39307 | 0.00000 | 65 | H | -2.47278 | -6.82618 | 4.64925 |
| 21 | C | -2.57061 | 5.61673 | 1.22283 | 64 | H | -2.66435 | -4.38673 | 4.08466 |
| 22 | C | -3.89463 | 6.08283 | 1.21749 | 63 | H | -1.50223 | -3.47009 | 2.06521 |
| 23 | C | -4.55708 | 6.31915 | 0.00000 | 56 | H | 2.05856 | -5.44043 | 2.18260 |
| 24 | C | -3.89463 | 6.08283 | -1.21749 | 96 | H | -2.54542 | 1.53791 | 2.11469 |
| 25 | C | -2.57061 | 5.61673 | -1.22283 | 97 | H | -3.41627 | 2.70178 | 4.15179 |
| 26 | C | 0.52854 | 6.76426 | 1.81961 | 98 | H | -5.87055 | 2.64151 | 4.68455 |
| 27 | C | 0.63920 | 5.39132 | 1.49522 | 99 | H | -7.44692 | 1.39815 | 3.17670 |
| 28 | C | 3.62501 | -1.51048 | -2.34476 | 100 | H | -6.58987 | 0.21355 | 1.16609 |
| 29 | C | 4.11427 | -2.16683 | -3.48738 | 55 | H | 4.41071 | -6.26787 | 2.17345 |
| 30 | C | 5.48626 | -2.13123 | -3.78611 | 54 | H | 5.59454 | -6.69119 | 0.00000 |
| 31 | C | -4.59503 | -1.71170 | 0.00000 | 53 | H | 4.41071 | -6.26787 | -2.17345 |
| 32 | C | -4.85347 | -2.37645 | -1.22076 | 52 | H | 2.05856 | -5.44043 | -2.18260 |
| 33 | C | -5.38211 | -3.67777 | -1.21614 | 45 | H | -4.66004 | -1.87068 | 2.18091 |
| 34 | C | -5.64968 | -4.33004 | 0.00000 | 44 | H | -5.59689 | -4.18204 | 2.17240 |

| | | | | | | | | | |
|---|---|---|---|---|---|---|---|---|---|
| 35 | C | -5.38211 | -3.67777 | 1.21614 | 107 | H | 6.58981 | -0.21351 | 1.16622 |
| 36 | C | -4.85347 | -2.37645 | 1.22076 | 108 | H | 7.44691 | -1.39877 | 3.17642 |
| 37 | C | 6.37044 | -1.43416 | -2.94220 | 109 | H | 5.87042 | -2.64170 | 4.68450 |
| 38 | C | 5.88597 | -0.77291 | -1.80317 | 110 | H | 3.41620 | -2.70223 | 4.15151 |
| 39 | C | 4.50630 | -0.80933 | -1.49319 | 111 | H | 2.54530 | -1.53766 | 2.11483 |
| 40 | C | 3.62501 | -1.51048 | 2.34476 | 43 | H | -6.07333 | -5.34751 | 0.00000 |
| 41 | C | 4.11427 | -2.16683 | 3.48738 | 42 | H | -5.59689 | -4.18204 | -2.17240 |
| 42 | C | 1.89620 | -5.39297 | 0.00000 | 41 | H | -4.66004 | -1.87068 | -2.18091 |
| 43 | C | 2.57060 | -5.61664 | -1.22283 | 34 | H | -2.05861 | 5.44041 | -2.18260 |
| 44 | C | 3.89464 | -6.08267 | -1.21749 | 33 | H | -4.41065 | 6.26817 | -2.17345 |
| 45 | C | 4.55708 | -6.31902 | 0.00000 | 32 | H | -5.59458 | 6.69122 | 0.00000 |
| 46 | C | 3.89464 | -6.08267 | 1.21749 | 118 | H | 6.58981 | -0.21351 | -1.16622 |
| 47 | C | 2.57060 | -5.61664 | 1.22283 | 119 | H | 7.44691 | -1.39877 | -3.17642 |
| 48 | C | 5.48626 | -2.13123 | 3.78611 | 120 | H | 5.87042 | -2.64170 | -4.68450 |
| 49 | C | 6.37044 | -1.43416 | 2.94220 | 121 | H | 3.41620 | -2.70223 | -4.15151 |
| 50 | C | 5.88597 | -0.77291 | 1.80317 | 122 | H | 2.54530 | -1.53766 | -2.11483 |
| 51 | C | 4.50630 | -0.80933 | 1.49319 | 31 | H | -4.41065 | 6.26817 | 2.17345 |
| 52 | C | -5.88602 | 0.77278 | 1.80317 | 30 | H | -2.05861 | 5.44041 | 2.18260 |
| 53 | C | -0.63920 | -5.39119 | 1.49522 | 23 | H | 4.66010 | 1.87061 | 2.18091 |
| 54 | C | -1.41434 | -4.54167 | 2.31338 | 22 | H | 5.59701 | 4.18195 | 2.17240 |
| 55 | C | -2.06940 | -5.05792 | 3.44419 | 21 | H | 6.07348 | 5.34740 | 0.00000 |
| 56 | C | -1.96057 | -6.42227 | 3.76068 | 20 | H | 5.59701 | 4.18195 | -2.17240 |
| 57 | C | -1.19093 | -7.27353 | 2.94688 | 129 | H | -0.08517 | 7.44109 | 1.20364 |
| 58 | C | -0.52858 | -6.76413 | 1.81960 | 130 | H | 1.09971 | 8.34375 | 3.19445 |
| 59 | C | -6.37049 | 1.43382 | 2.94233 | 131 | H | 2.47284 | 6.82635 | 4.64919 |
| 60 | C | -5.48635 | 2.13094 | 3.78622 | 132 | H | 2.66453 | 4.38692 | 4.08454 |
| 61 | C | -4.11436 | 2.16660 | 3.48750 | 133 | H | 1.50230 | 3.47025 | 2.06517 |
| 62 | C | -3.62510 | 1.51046 | 2.34476 | 19 | H | 4.66010 | 1.87061 | -2.18091 |
| 63 | C | -4.50636 | 0.80927 | 1.49318 | 140 | H | -0.08517 | 7.44109 | -1.20364 |
| 64 | C | -0.63920 | -5.39119 | -1.49522 | 141 | H | 1.09971 | 8.34375 | -3.19445 |
| 65 | C | -1.41434 | -4.54167 | -2.31338 | 142 | H | 2.47284 | 6.82635 | -4.64919 |
| 72 | C | -2.06940 | -5.05792 | -3.44419 | 143 | H | 2.66453 | 4.38692 | -4.08454 |
| 78 | H | 0.08506 | -7.44099 | -1.20359 | 9 | P | 0.19129 | -4.72376 | 0.00000 |
| 144 | H | 1.50230 | 3.47025 | -2.06517 | 11 | P | -3.83701 | -0.03542 | 0.00000 |
| 10 | P | -0.19130 | 4.72389 | 0.00000 | 12 | P | 3.83700 | 0.03539 | 0.00000 |

$[Au_4]^{2+}$ flat:

BP86/def2-SV(P)//PBE0-D4/def2-TZVP

Total energy: -542.25841386436 H

HOMO-LUMO: 3.616 eV

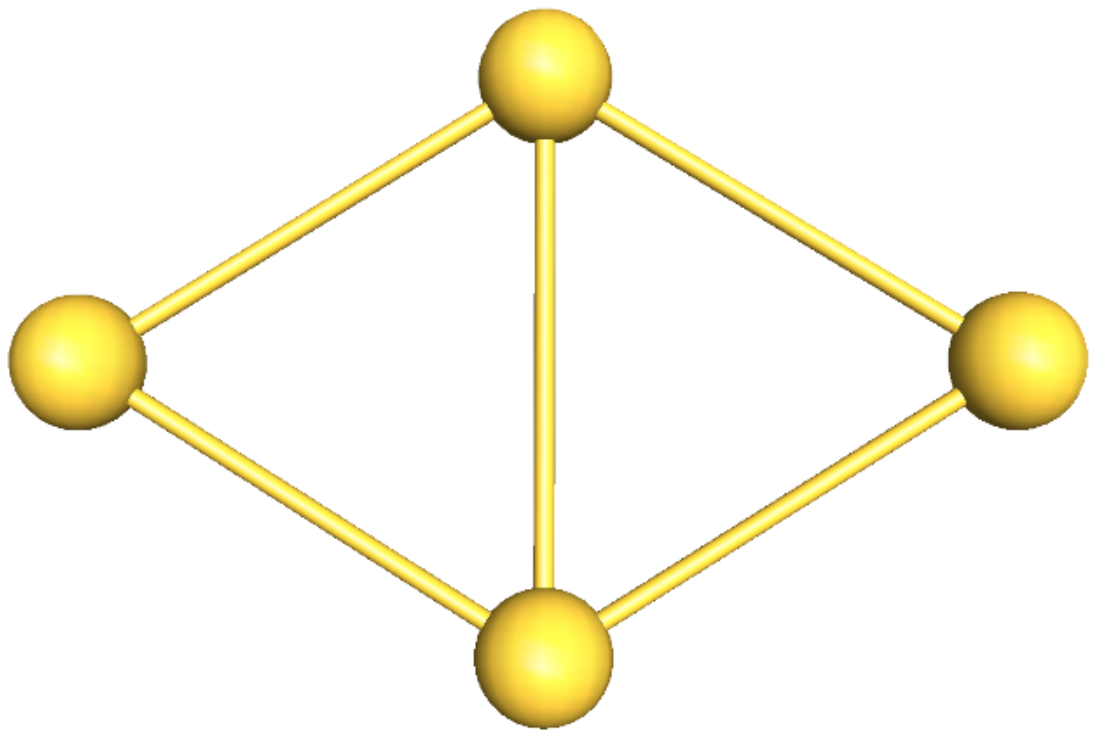

*Figure S 5: Image of the minimum structure of the planar $[Au_4]^{2+}$.*

*Table S 6: Atomic coordinates of the planar $[Au_4]^{2+}$.*

| Atom number | Atom symbol | x | y | z | Atom number | Atom symbol | x | y | z |
|---|---|---|---|---|---|---|---|---|---|
| 2 | Au | 0.00000 | -1.41619 | 0.00000 | 4 | Au | -2.27600 | 0.00000 | -0.00000 |
| 3 | Au | 2.27600 | 0.00000 | -0.00000 | 1 | Au | 0.00000 | 1.41619 | 0.00000 |

## $[Au_4]^{2+}$ tetrahedron:

BP86/def2-SV(P)//PBE0-D4/def2-TZVP

Total energy: -542.28073610003 H

HOMO-LUMO: 4.954 eV

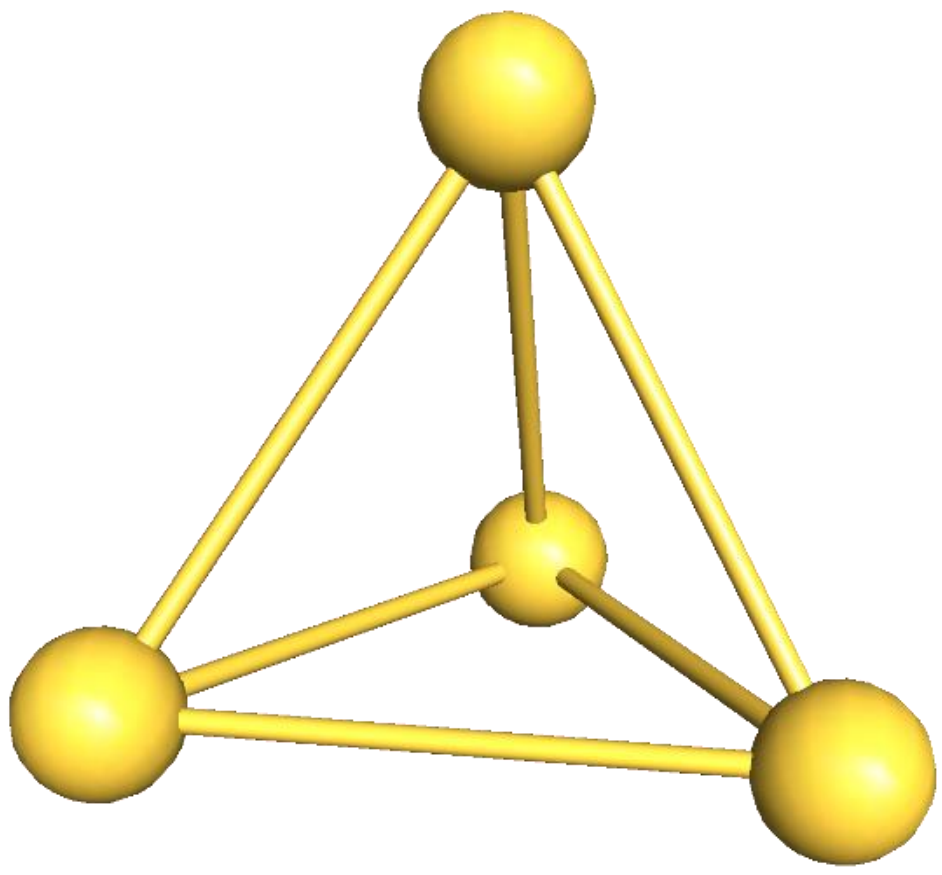

*Figure S 6: Image of the minimum structure of the tetrahedral $[Au_4]^{2+}$.*

*Table S 7: Atomic coordinates of the tetrahedral $[Au_4]^{2+}$.*

| Atom number | Atom symbol | x | y | z | Atom number | Atom symbol | x | y | z |
|---|---|---|---|---|---|---|---|---|---|
| 2 | Au | -0.97060 | -0.97060 | -0.97060 | 4 | Au | -0.97060 | 0.97060 | 0.97060 |
| 3 | Au | 0.97060 | -0.97060 | 0.97060 | 1 | Au | 0.97060 | 0.97060 | -0.97060 |

### 3.2 Calculations with Orca

DFT and TDDFT calculations were performed in ORCA 6.[16-21] The ground state geometry were preoptimized using BP86 [7, 8] or PBE0 [9] and Grimme's D4 dispersion correction [10-12] with Def2-TZVP [13] basis sets with the appropriate auxiliary basis set (Def2/J)[14] and for Au the adequate effective core potentials (Def2-ECP).[15] For the final geometry optimization and for the TDDFT calculations, the hybrid functional PBE0 was employed alongside the D4 dispersion correction, while scalar relativistic effects were accounted for using the eXact Two-Component (X2C) Hamiltonian [22, 23]. All calculations with X2C Hamiltonian, used the diagonal local approximation to the unitary transformation matrix (DLU approximation) within the X2C calculation as well as a gaussian finite nucleus model [24] and the correction for picture change effects for gradients and properties. Furthermore, the triple-zeta basis set optimized for X2C (X2C-TZVPPALL) [25, 26] and its corresponding auxiliary set (X2C/J) [25, 26] were used. Spin–orbit coupling in TDDFT calculations were treated with the spin-orbit mean-field method RI-SOMF(1X)[27] In all calculations, strict Self-Consistent Field (SCF) convergence criteria TightSCF were used, for geometry optimization the convergence criteria were also set to TightOPT. For the all-electron X2C calculations with X2C-TZVPPALL basis set often achieving the SCF convergence is an issue. In these cases, the trust-region augmented Hessian method (TRAH) [20] was used as sole SCF convergence algorithm and the number of SCF iteration steps was increased to 500.

As initial input geometry a structure from single crystal x-ray diffraction was used. For TDDFT calculations of the spectra typically the 50 lowest singlet and triplet excided states/transitions (50 roots) were requested, corresponding to 200 transitions in the spin-orbit coupled TDDFT calculation.

# 4. Optical measurements

## 4.1. Luminescence measurements in solution and powder samples (broadband excitation)

For all measurements in solution, a stock solution of 27 mg $[Au_4(dppb)_3][InCl_4][InCl_4(THF)]$ dissolved in 10 mL acetonitrile Is prepared. 0.5 mL of this solution are diluted with 9.5 mL acetonitrile.

The absorption measurements were done on a T60 UV/Vis-spectrometer from PG Instruments in a 1 cm quartz glass cuvette.

Steady-state spectra were measured on a Horiba Fluorolog-3 spectrofluorimeter equipped with a 450 W xenon lamp for steady-state measurements. For fluorescence liftimes, a pulsed Laser (PicoQuant LDH-P-C-375, $\lambda_{exc}$ = 371±2 nm, pulse width ca. 50 ps FWHM, $P_{max,avg} \approx 5$ mW) was used in combination with the Techspec® (Edmund optics) shortpass filter cutoff 475 nm (OD>4.0). Emitted light was detected by a Hamamatsu R13456 PMT (UV/vis/NIR, 200 nm < $\lambda_{em}$ < 1000 nm). A double grating monochromator 320DFX (1200 grooves/nm, blazed at 330 nm or 600 grooves/nm, blazed at 750 nm) was used for spectral selection in the excitation path, while in the emission path the single grating monochromator iHR550 (1200 grooves/mm, blazed at 500 nm; 950 grooves/mm, blazed at 900 nm; 1800 grooves/mm, blazed at 500 nm) was used. To avoid higher order excitation light, long pass filter glass plates (Schott, 3 mm thickness) were used in the emission beam path when needed. The shown spectra are always corrected for the sensitivity of the spectral and detection system as well as for the illumination intensity.

For steady state spectra or lifetime measurements (TCSPC) the solution and solid samples (powers) were prepared in regular borosilicate glass NMR tubes (5 mm outer diameter) and measured in a homemade tube holder. If not stated otherwise all spectra were recorded at room temperature (20 – 25°C). Lifetime data analysis (deconvolution, statistical parameters, etc.) was performed using

the software package DAS analysis from Horiba. The decay data were analyzed by tail-fitting (exclusion of the lamp-puls region). For the lifetimes from powder sample of **1** (Tab. S8), different fits and measurement windows were used: T1 and T2 (77 K and RT) single pulse excitation, 100 ns window fitted biexponentially. T3, T4 and T5 (77 K) multi pulse excitation, 360 µs window fitted triexponentially. T3, T4 and T5 (RT) multi pulse excitation, 50 µs window, fitted biexponentially. The Xisquare values range between 0.978 and 1.249.

Measurements at 77 K were performed in regular borosilicate glass NMR tubes (5 mm outer diameter). For cooling and spectrum detection the sample tube within a cold finger quartz dewar (Horiba K-158) filled with liquid nitrogen was placed into the light path.

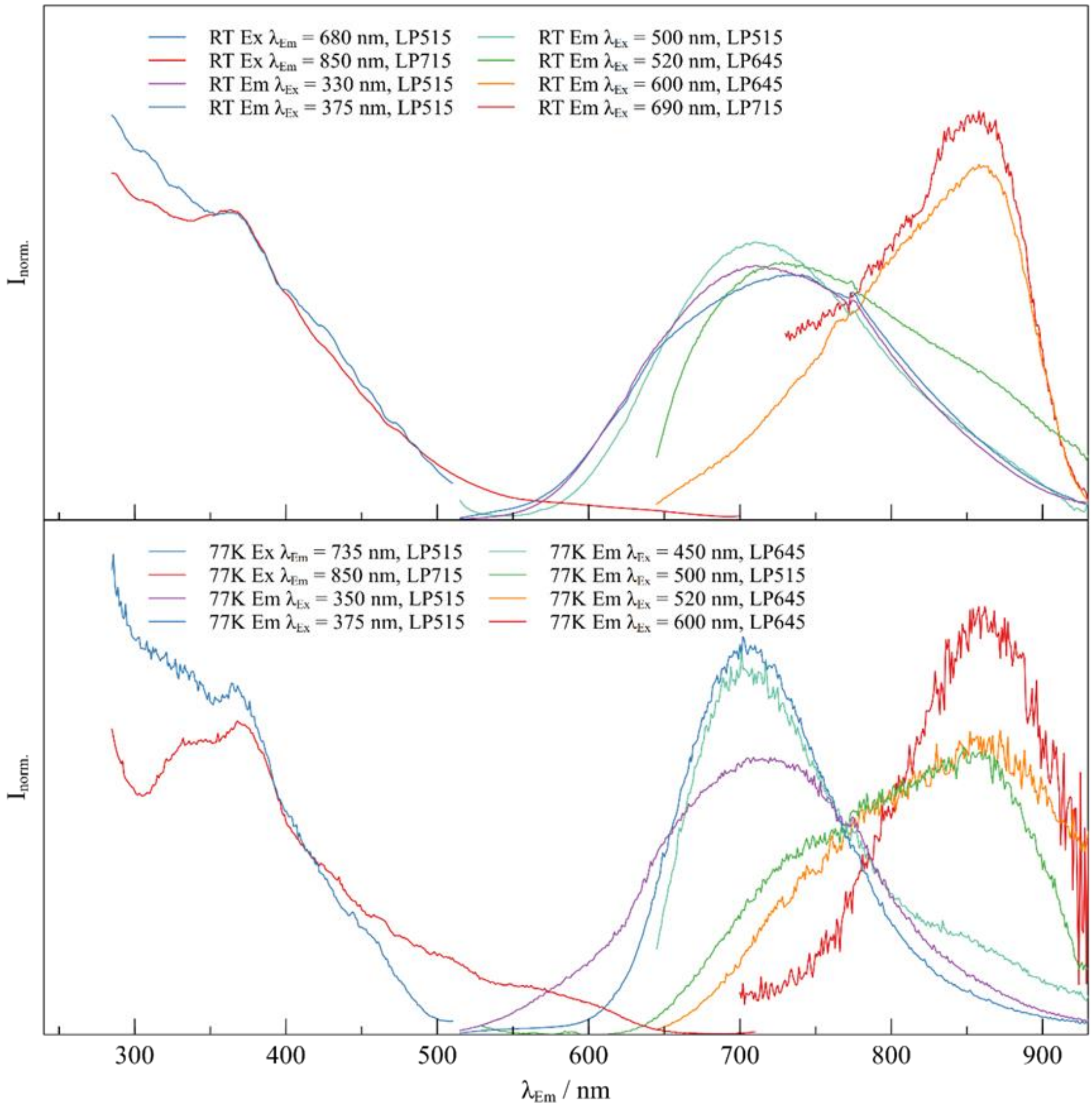


*Figure S 7: Excitation and emission spectra (area normalized) of a powder sample of **1** at RT (top) and 77 K (bottom). The The longpass filters used to separate the excitation light from the emission are indicated as LP515, LP645 and LP715 defining the cut off wavelength. At 77 K, the spectrum after the excitation at 600 nm is only plotted from 700 nm for clarity as no spectral features appear blue of this wavelength.*

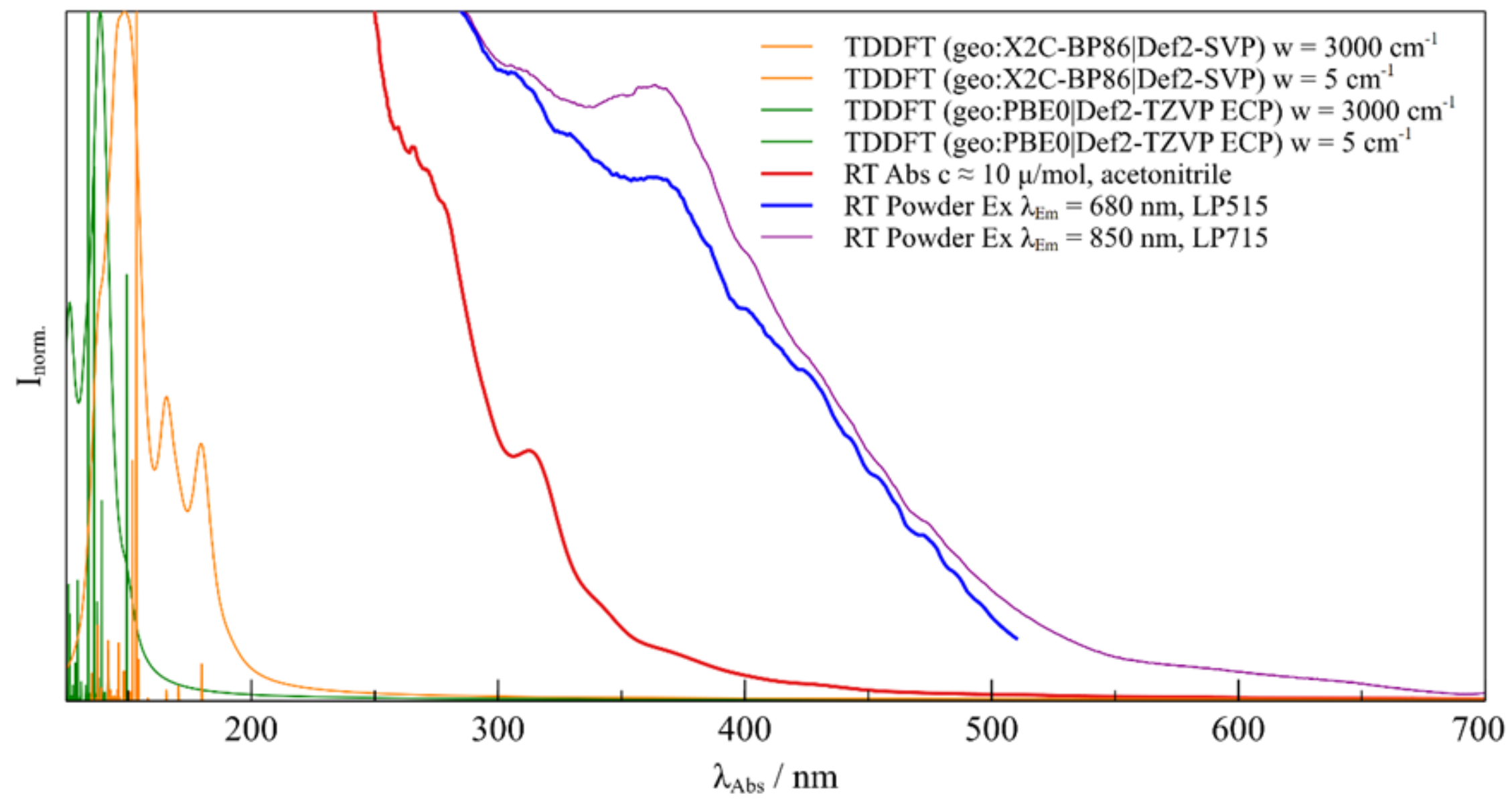


*Figure S 8: Experimental absorption (solution) and excitation (powder) spectra in comparison to the calculated spectra obtain from TDDFT calculations (X2C-PBE0|TZVPP, 200 spin-orbit coupled transitions, with different Lorentzian line broadening w) for two differently optimized geometries (normalized maximum intensity).*

*Table S 8: Lifetimes from powder sample of **1** in ns after illumination at 375 and 520 nm, measured at RT and 77 K. T1 and T2 (77 K and RT) single pulse excitation, 100 ns window, biexp. fit, T3, T4 and T5 (77 K) multi pulse excitation, 360 µs window, triexp. fit and T3, T4 and T5 (RT) multi pulse excitation, 50 µs window, biexp. fit.*

| **$\lambda_{Ex}$ = 520 nm** | | **T1** | | **T2** | | **T3** | | **T4** | | **T5** | |
|---|---|---|---|---|---|---|---|---|---|---|---|
| **RT** | **650 nm** | 0.54 | 99 % | | | 1920 | 12 % | 4530 | 88 % | | |
| | **890 nm** | | 88 % | | | | 70 % | | 30 % | | |
| **77 K** | **650 nm** | 0.68 | 99 % | 7.3 | 1 % | 3430 | 12 % | 11300 | 29 % | 24100 | 12 % |
| | **890 nm** | | 87 % | | 13 % | | 32 % | | 69 % | | 3 % |
| **$\lambda_{Ex}$ = 375 nm** | | **T1** | | **T2** | | **T3** | | **T4** | | **T5** | |
| **RT** | **590 nm** | | | 4.7 | 63 % | 1680 | 27 % | 4250 | 73 % | | |
| | **890 nm** | | | | 32 % | | 34 % | | 66 % | | |
| **77 K** | **590 nm** | | | 2.3 | 93 % | 2660 | 38 % | 6170 | 50 % | 22800 | 13 % |
| | **890 nm** | | | | 59 % | | 19 % | | 74 % | | 7 % |

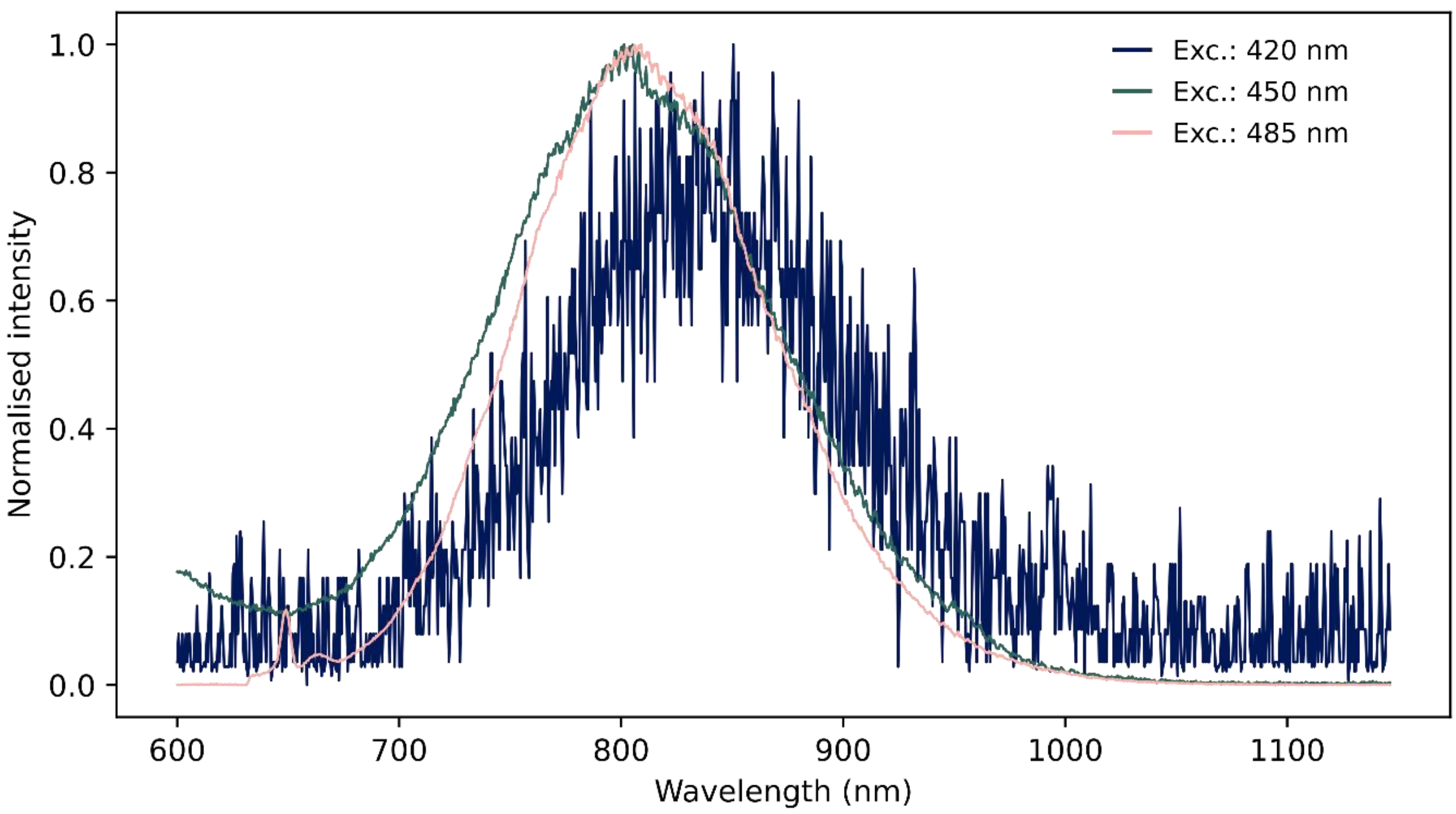


*Figure S 9: Normalized emission spectra of **1** in solution (acetonitrile) under different excitation wavelengths.*

## 4.2. Luminescence under focused laser excitation

### 4.2.1 Single crystalline sample

A sample of single crystals of $[Au_4(dppb)_3][InCl_4][InCl_4(THF)]$ was transferred on a Si-waver which was placed on the scanning stage of the custom-built low temperature laser scanning confocal microscope. The excitation light from a white light source (SuperK Fianium FIU15 NKT Photonics) was filtered by a tunable bandpass filter (LLTF Photon etc.) allowing continuous tuning of the excitation wavelength. The light was guided into the Jannis SVT 350 varitemp bath cryostat and focused onto the sample by a NA 0.85 objective lens. The emitted light is spectrally selected by a fitting longpass filter (LPF), passes the second pinhole in a non-expanding telescope and is guided either to the spectrometer (Andor Si500 Spectrograph with 200 l/mm Blaze 720 nm grating and a Andor CCD cooled electronically to -70 °C) or the APD (Micro Photon Device avalanche photodiode). TCSPC data analysis and fitting (Xisqaure between 0.936 to 1.071) was performed with the Symphotime 64 software (PicoQuant) in the n-exponential tailfitting model keeping the fitting boundaries constant over all measurements.

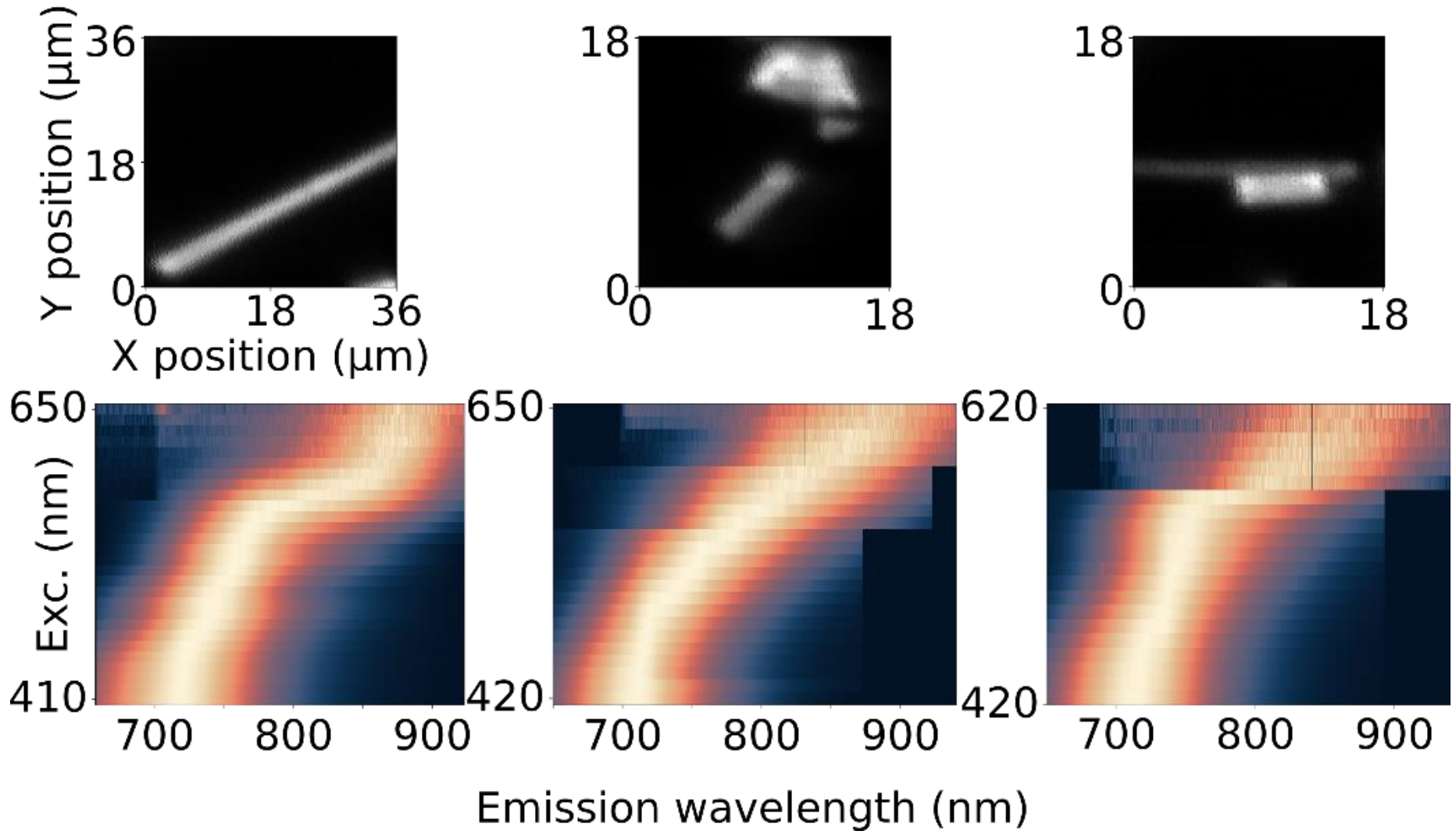


*Figure S 10: All three measured (77 K) single crystals are presented as a scan image (top) and with corresponding emission spectra under excitation tuning (bottom) all measured crystals show the same trend of excitation dependent emission. Discrete steps in intensity are caused by adjusting the grating and longpass filters for optimal coverage of the emission spectrum of the crystals.*

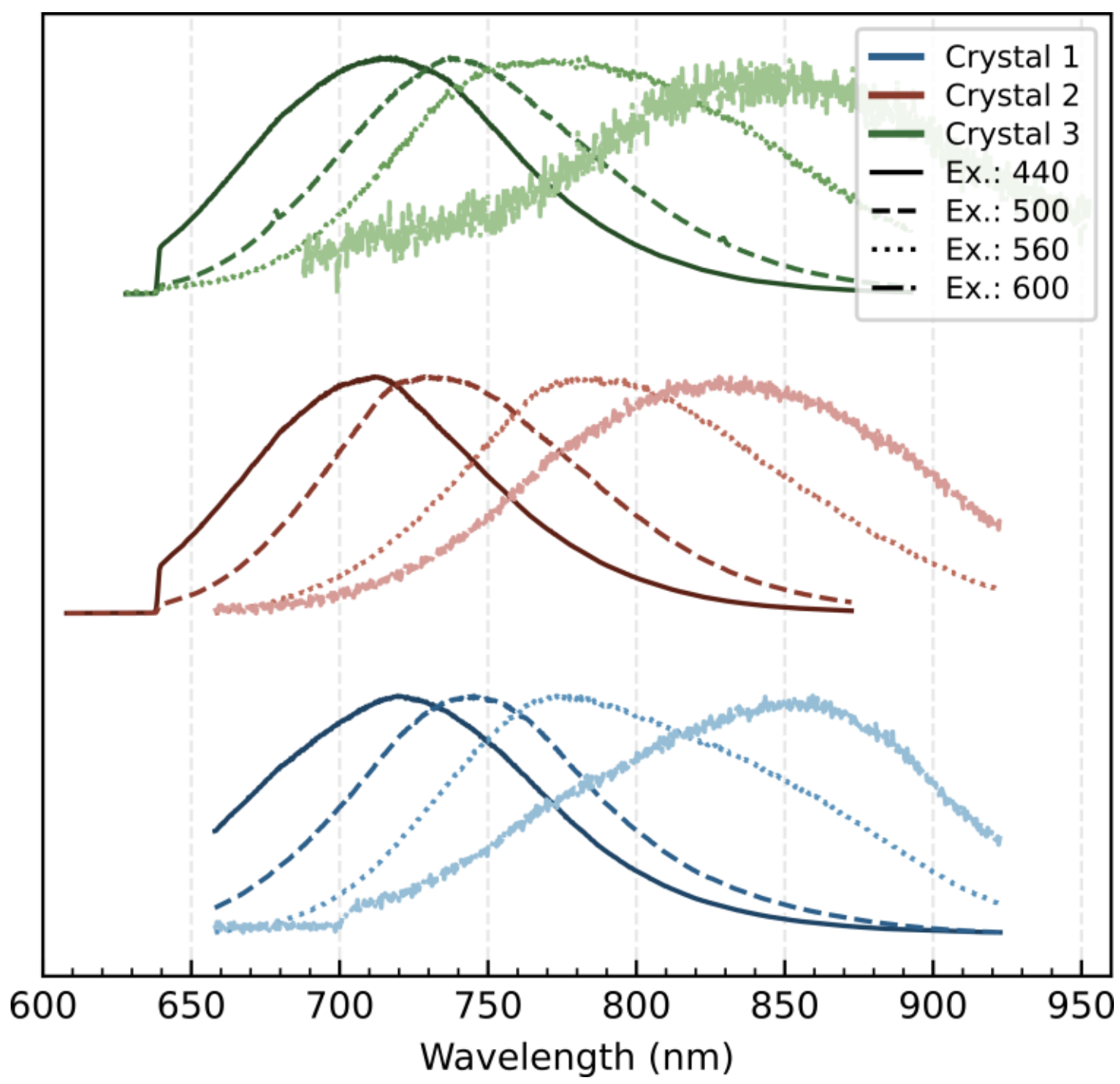


*Figure S 11: Representative spectra of the three single crystals measured at the same excitation wavelengths given in the legend.*

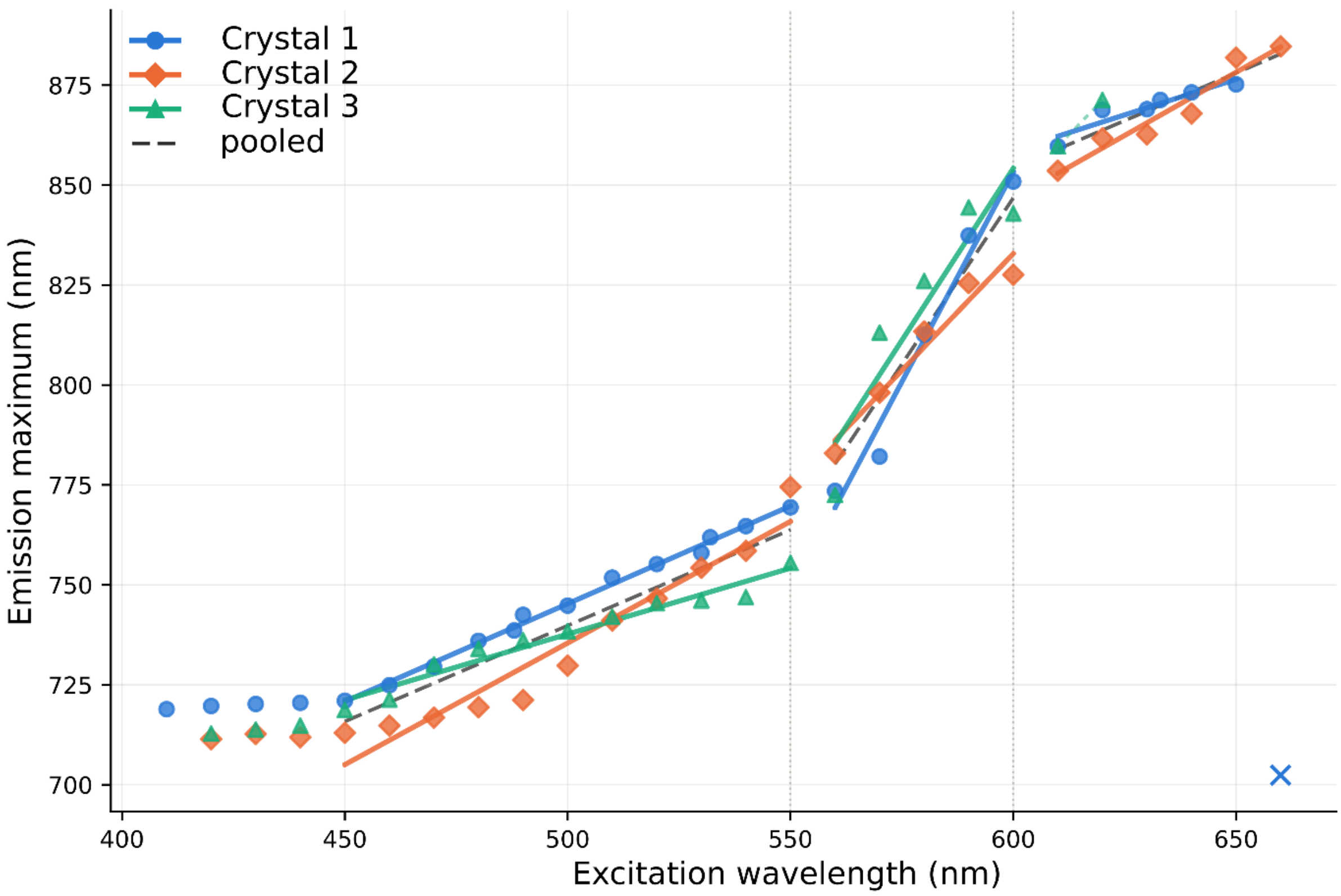


*Figure S 12: Emission maximum determined after 5 nm floating average smoothing of the spectra against the excitation wavelength for all three single crystals at liquid nitrogen temperature. Sections wise fitted with linear regression of each crystal as well as the pooled data of all crystals.*

*Table S 9: Parameters of the linear regression on the single crystal data shown in figure S12.*

| Group | n | Slope (nm/nm) | Slope | Intercept | Pearson r | R² |
|---|---|---|---|---|---|---|
| SC1 | 13 | 0,489 | 0,021 | 500,7 | 0,997 | 0,995 |
| SC2 | 11 | 0,608 | 0,102 | 431,3 | 0,969 | 0,938 |
| SC3 | 11 | 0,33 | 0,046 | 572,6 | 0,978 | 0,956 |
| **All SC (pooled)** | **35** | **0,48** | **0,07** | **500** | **0,92** | **0,847** |
| SC1 | 5 | 2,101 | 0,389 | -407,3 | 0,987 | 0,974 |
| SC2 | 5 | 1,168 | 0,304 | 132,1 | 0,975 | 0,95 |
| SC3 | 5 | 1,721 | 0,798 | -178,4 | 0,925 | 0,856 |
| **All SC (pooled)** | **15** | **1,663** | **0,375** | **-151,2** | **0,924** | **0,853** |
| SC1 | 6 | 0,357 | 0,131 | 644,7 | 0,936 | 0,876 |
| SC2 | 6 | 0,632 | 0,156 | 467,2 | 0,97 | 0,941 |
| SC3 | 2 | - | - | - | - | |
| **All SC (pooled)** | **14** | **0,476** | **0,14** | **568,3** | **0,888** | **0,788** |

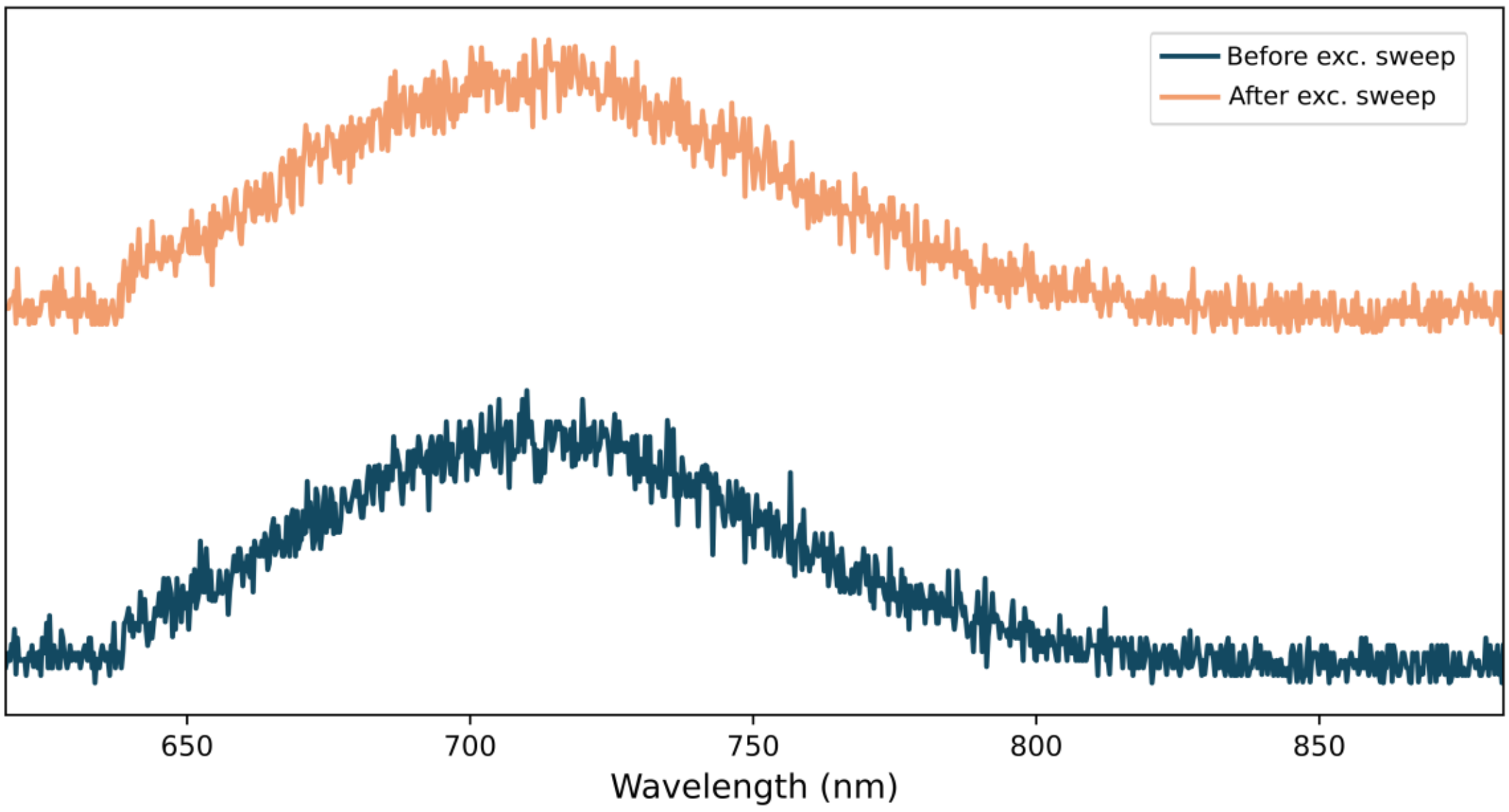


*Figure S 13: Normalized emission spectra under short wavelength excitation (400 nm) before (blue) and after (orange) the excitation wavelength sweep.*

*Table S 10: Average Parameters from the biexponential TCSPC fit $I = A_1 \exp(1/\tau_1)+A_2 \exp(1/\tau_2)$ of all three crystals under changing excitation wavelengths. $\tau_i$ is the resulting lifetime with the corresponding amplitude $A_i$. $A_2/A_1$ gives the relative contribution of each lifetime term, showing that for short wavelength excitation the long lifetime contribution $\tau_1$ is dominating while for long wavelength excitation the shorter $\tau_2$ is dominant.*

| ExcWL (nm) | $\tau_1$ (ns) | $\tau_2$(ns) | $A_2/A_1$ |
|---|---|---|---|
| 420 | 4457 | 1394 | 0.0012 |
| 430 | 4493 | 1684 | 0.0026 |
| 440 | 4449 | 324 | 0.0277 |
| 450 | 4148 | 4 | 0.1610 |
| 460 | 4304 | 159 | 0.0325 |
| 470 | 5214 | 630 | 0.1020 |
| 480 | 4060 | 9 | 0.1713 |
| 488 | 4163 | 7.2 | 0.1149 |
| 490 | 4103 | 55 | 0.1089 |
| 500 | 4505 | 405 | 0.0686 |
| 510 | 3987 | 282 | 0.0679 |
| 520 | 3687 | 97 | 0.0963 |
| 530 | 3696 | 135 | 0.2140 |
| 540 | 3696 | 139 | 0.2658 |

| | | | |
|---|---|---|---|
| 550 | 3487 | 142 | 0.4545 |
| 560 | 3432 | 164 | 0.8461 |
| 570 | 2470 | 119 | 0.9842 |

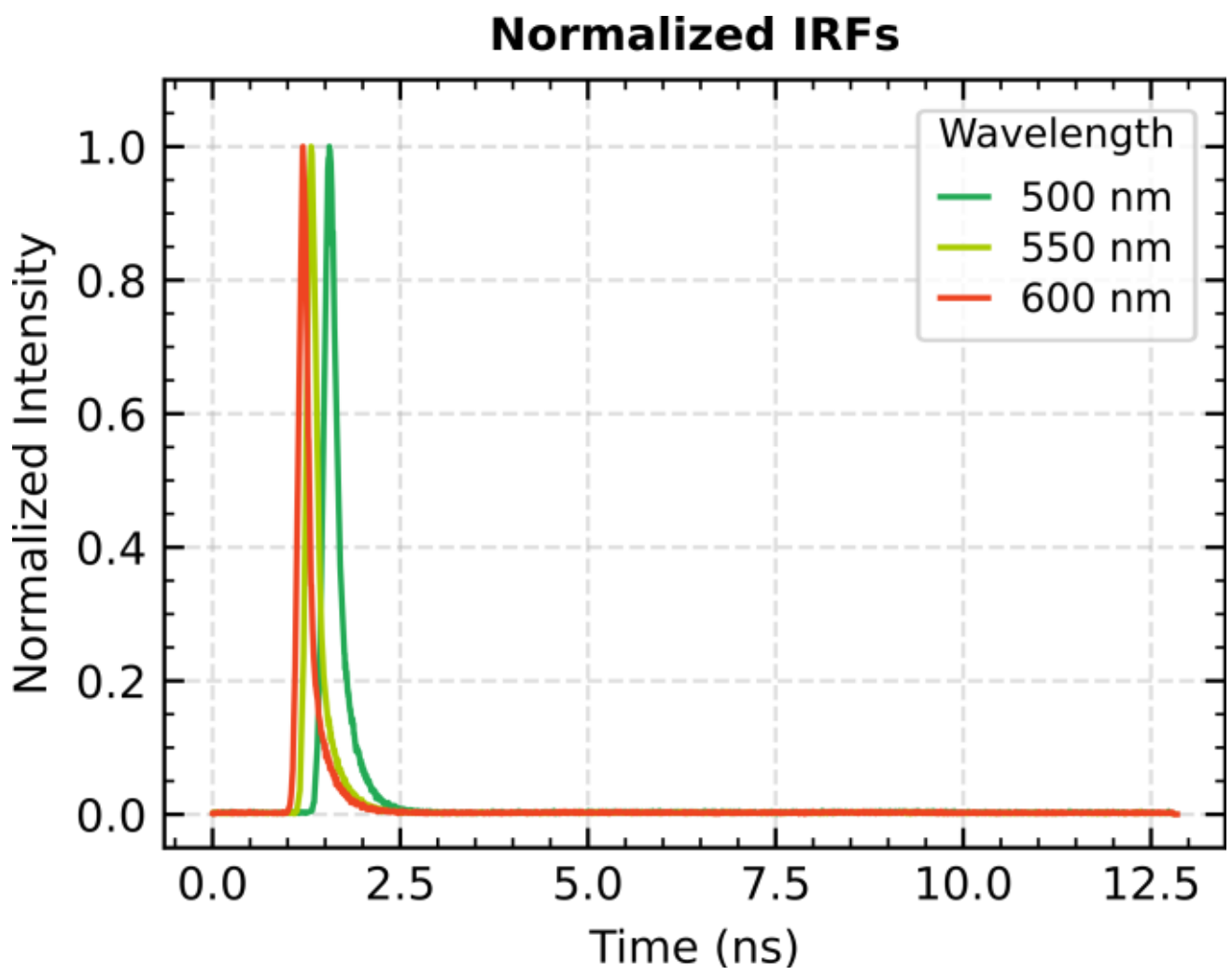


*Figure S 14: IRF of the tunable laser source at 80MHz repetition rate at three different wavelengths detected in reflection in the confocal scanning setup. Note that an IRF measurement at 150 kHz is not possible as the time resolution is limited to 1 ns per bin at this frequency leading to the IRF would be covered by one time bin.*

### 4.2.2 Powder sample

The measurements were performed as in 4.2 with a blended sample of $[Au_4(dppb)_3][InCl_4][InCl_4(THF)]$.

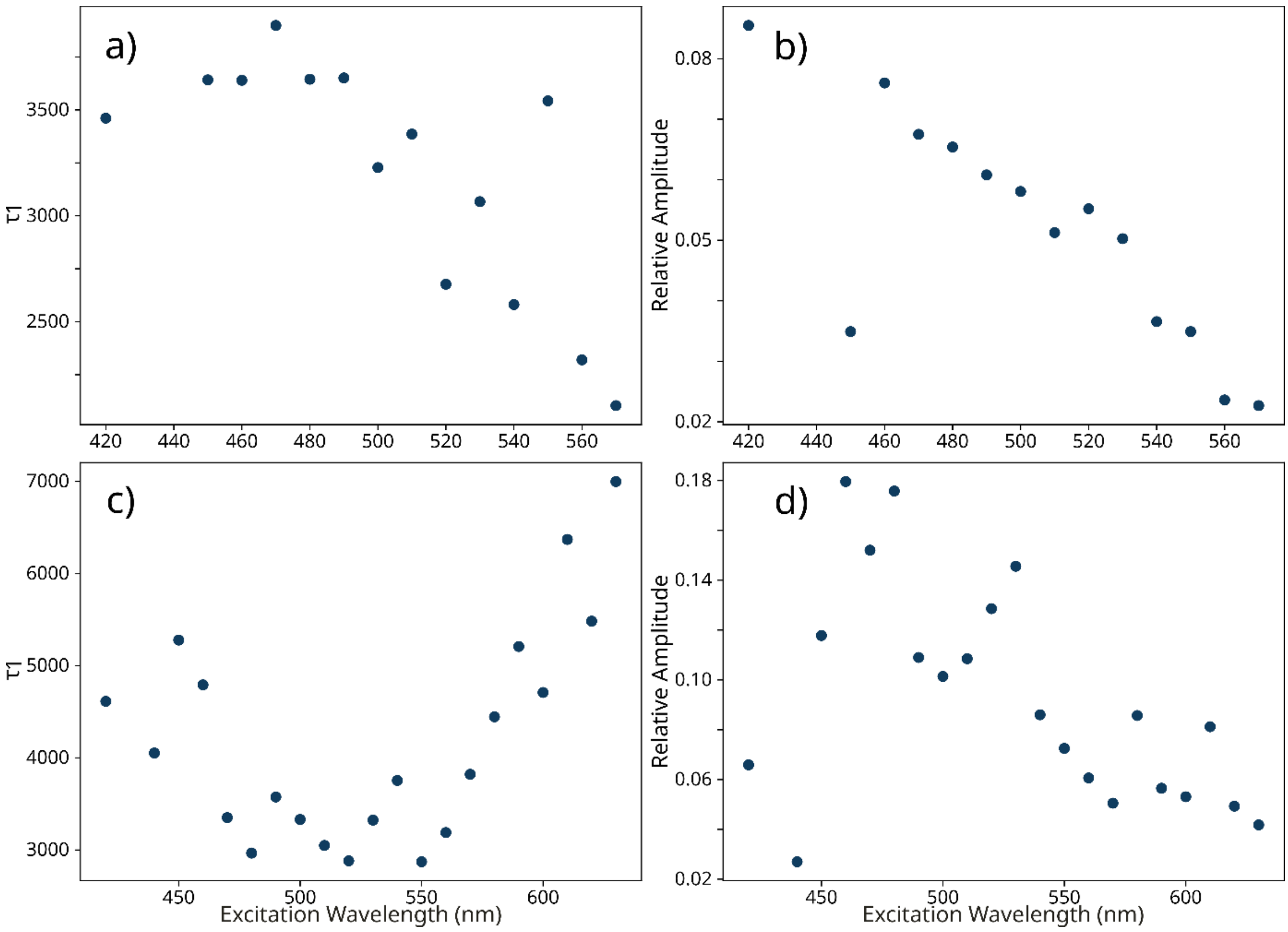


*Figure S 15: Long lifetime contribution $\tau_1$ and its and relative Amplitude (A1/A2) in the biexponentially fitted data plotted against the excitation wavelength. a) Long lifetime contribution under different excitation wavelengths at RT. b) Relative amplitude A1/A2 under different excitation wavelengths at RT. c) Long lifetime contribution under different excitation wavelengths at 77 K. d) Relative amplitude A1/A2 under different excitation wavelengths at 77 K.*

### 4.3. Quantum Yield

For the quantum yield measurements at room temperature, a calibrated 3.2" integrating sphere K Sphere-Petite (Horiba) was used. In the sphere, the sample is directly illuminated by the excitation light and a PTFE block in the direction of the emission monochromator avoids directly scattered or reflected light reaching the emission path. The wavelength depended correction for the integrating sphere was obtained by comparing the spectrum of the excitation lamp with a spectralone diagonal half cuvette (Horiba) in the sample position with and without the surrounding sphere. Typically, the sample powder is adsorbed to a 2-3 cm piece of a magnesia rod which then is placed in a PTFE capped quartz tube (4 mm outer diameter) for the measurement. As pure scattering sample (reference) a 2 -3 cm piece of clean magnesia rod in a 4 mm quartz tube was prepared in the same way. In all measurements, the samples were excited with the given excitation-wavelength and an excitation band pass of at least 4 nm, using the emission band pass to avoid overloading the detector for the pure scattering reference. The Rayleigh scattering peak was measured either in the same spectrum as the luminescence band or in a separate spectrum using a calibrated orifice prior the detector (8-12 % transmission). If necessary, to avoid higher order excitation light, long pass filter glass plates (Schott, 3 mm thickness) were used in the emission beam path and the resulting emission spectra were corrected using the transmission spectrum of the respective filter. To validate the determinations, the measurement was repeated at least three times, and the result is obtained as average value with an uncertainty estimated to ≤ 25 %. To avoid systematic errors, periodically the measurement is cross-referenced to known quantum yield standards as quinine sulfate (in 0.1 N $H_2SO_4$, $\phi = 0.50$)[28] , rhodamine6G (ethanol, $\phi = 0.95$)[29] or Ru(bipy)$_3$ (water, air, $\phi = 0.040$)[30].

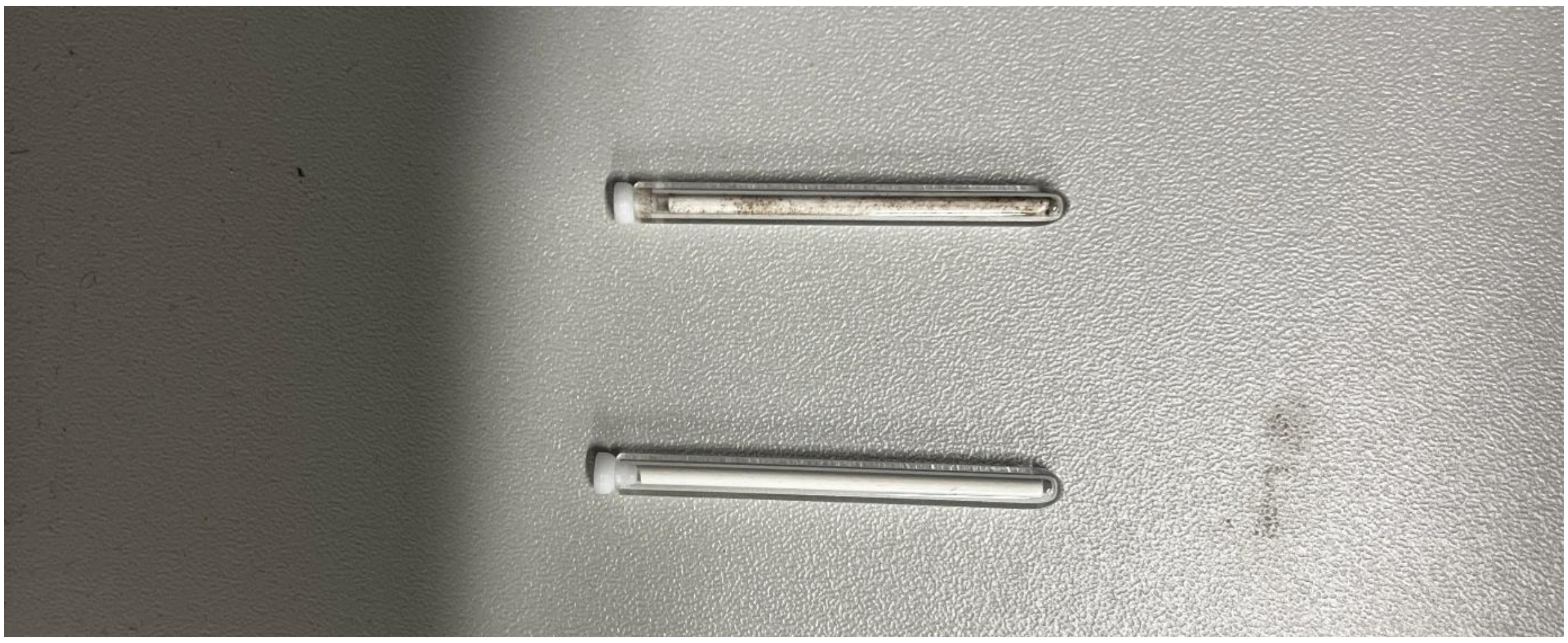

*Figure S 16: Image sample and reference in a PTFE capped quartz tube. The $[Au_4(dppb)_3][InCl_4][InCl_4(THF)]$ cluster adsorbed to a piece of magnesia rod (top) and the reference magnesia rod without cluster (bottom).*

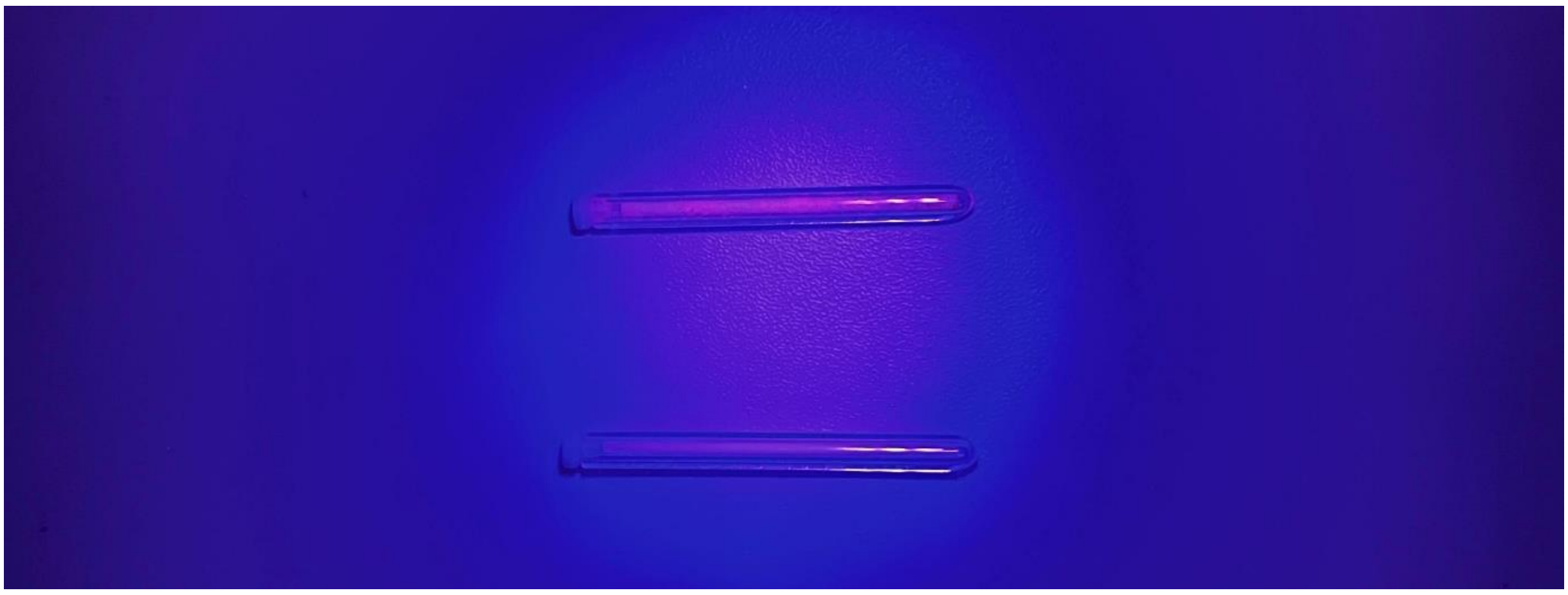

*Figure S 17: The $[Au_4(dppb)_3][InCl_4][InCl_4(THF)]$ cluster adsorbed to a piece of magnesia rod (top) and the reference magnesia rod without cluster (bottom) under illumination with UV-light (395 nm).*

# 5. Crystallography

Crystals of $[Au_4(dppb)_3][InCl_4][InCl_4(THF)]$ were mounted on the diffractometer at 150 K. The data were collected on a Rigaku XtaLAB Synergy-S X-ray diffractometer for single crystal X-ray diffractometer equipped with a PhotonJet-S microfocus sealed tube for monochromated MoKα radiation (λ = 0.71073 Å) and equipped with an Oxford Cryosystems cryostat. A semiempirical absorption correction using spherical harmonics was applied using the SCALE3 ABSPACK algorithm. The structure was solved by direct methods and refined against $F^2$ for all observed reflections. Programs used: SHELXT and SHELXL [31, 32] within the Olex2 program package.[33]

The SQUEEZE routine was used to identify four additional voids containing 38 electrons each (Volume 131 $Å^3$ each), fitting in good accordance to one thf molecule per void.

Structure refinement shows some disordering issues concerning the modeling of the anions $[(thf)InCl_4]^-$ and $[InCl_4]^-$. The solvent-free anion $[InCl_4]^-$ could be best described as a 94:6 disordered model. The Lewis adduct $[(thf)InCl_4]^-$ shows the same 94:6 disorder for the $InCl_4$ fragment. The thf molecule coordinated to the major domain (94%) is additionally disordered in a 60:40 ratio and could be modeled this way. The distortion of the $InCl_4$ fragment in the 6% minor domain also indicates a Lewis base coordination to the In atom, but since the very low occupancy of 6% and the presence of a thf molecule in the major domain almost at the same position, which is already modeled as disordered, a refinement of a 6% thf molecule is not reasonable. Therefore, the disordered thf molecule in the 94% major domain was set as 100% occupancy in sum to get a reasonable sum formula. To get a reasonable structure model, the thermal ellipsoids were equalized using SIMU and RIGU restraints, also SADI was used to equalize the In-Cl distances, which is very important especially for the minor domain.

The H atom positions in all compounds were refined using a riding model. The supplementary crystallographic data (for CCDC numbers, see Table S ) can be obtained online free of charge at www.ccdc.cam.ac.uk/conts/retrieving.html or from Cambridge Crystallographic Data Centre, 12 Union Road, Cambridge CB21EZ; Fax: (+44)1223-336-033; or deposit@ccdc.cam.ac.uk.

*Table S 11: Crystal data.*

| | |
|---|---|
| Empirical formula | $C_{98}H_{88}Au_4Cl_8In_2O_2P_6$ |
| Formula weight | 2784.60 |
| Temperature/K | 149.99(10) |
| Crystal system | monoclinic |
| Space group | $P2_1/c$ |
| a/Å | 14.65060(10) |
| b/Å | 38.1674(4) |
| c/Å | 19.19610(10) |
| α/° | 90 |
| β/° | 107.4200(10) |
| γ/° | 90 |
| Volume/Å$^3$ | 10241.68(15) |
| Z | 4 |
| $\rho_{calc}$g/cm$^3$ | 1.806 |
| μ/mm$^{-1}$ | 6.499 |
| F(000) | 5328.0 |
| Crystal size/mm$^3$ | 0.172 × 0.099 × 0.037 |
| Radiation | Mo Kα (λ = 0.71073) |
| 2Θ range for data collection/° | 4.268 to 56.564 |
| Index ranges | $-19 \le h \le 19$, $-50 \le k \le 50$, $-25 \le l \le 25$ |
| Reflections collected | 140643 |
| Independent reflections | 25427 [$R_{int}$ = 0.0297, $R_{sigma}$ = 0.0235] |
| Data/restraints/parameters | 25427/216/1209 |
| Goodness-of-fit on $F^2$ | 1.111 |
| Final R indexes [I>=2σ (I)] | $R_1$ = 0.0341, $wR_2$ = 0.0655 |
| Final R indexes [all data] | $R_1$ = 0.0422, $wR_2$ = 0.0675 |
| Largest diff. peak/hole / e Å$^{-3}$ | 1.92/-1.53 |
| CCDC number | 2538607 |

# 6. Mechanistic discussion

For **1**, we hypothesize that the emission is neither that of a single, fully relaxed emitter nor a classical (azulene-type) higher-state anti-Kasha effect.[34] Following observations are supportive of this:

The excitation dependence increases through cooling. As Kasha–Vavilov behavior requires internal conversion and vibrational relaxation fast enough to funnel all populations into the lowest excited state before emission,[34] an effect that grows as these relaxation channels are slowed, indicates kinetically controlled branching.[35] A low-temperature intensity rise with multi-component emission is also documented for $Au_{25}$ and $Au_{38}$ clusters, where branching among components with state-specific electron–vibration coupling is reported.[36]

The decay resolves into at least five components spanning nanoseconds to microseconds where the fast decay processes could be assigned to fluorescence, the slow ones with triplet emission. Such multi-channel radiative behavior is established in gold clusters.[37] The component multiplicity implies conformer heterogeneity, consistent with the multi-exponential emission of multiple frozen conformers occupying slightly different sites in rigid glasses.[38] In a single crystal we expect this effect to be of limited influence due to the high purity and order of the compound.

A very short-lived far-red/NIR component appears only under red excitation at low temperature. Its low energy excludes a higher-state anti-Kasha origin, which would emit to the blue. Instead, it could indicate direct photo selection of a low-lying, weakly coupled state that is kinetically bypassed under blue excitation, where the upper states relax into the dominant, longer-lived channels. Its short lifetime hints on direct radiative decay rather than a slowly fed channel, and argues against a long-lived (µs–ms) triplet sublevel. Excitation-dependent lifetimes of exactly this kind, arising from a distribution of frozen environments, are long established, for example the excitation-wavelength-dependent phosphorescence lifetime of quinoline in rigid 77 K media.[39]

Beyond single-cluster photophysics, collective effects in the crystalline state could also contribute. In gold systems the emission energy is shown to depend directly on the Au–Au distance,[40, 41] a single crystal presenting a distribution of aurophilic (Au···Au) contacts can host multiple of collective, low-energy emissive states that different excitation energies address selectively, a mechanism already shown leading to excitation-dependent emission in assembled gold clusters with varied Au(I)···Au(I) distances.

To summarize, in literature we find examples for similar optical properties linked to slow internal conversion, different triplet sublevels, vibronic trapping, crystal-site heterogeneity and also aurophilic interactions in different systems. Due to the limitations of our measurements aimed at the phenomenological description of this new compound and its fascinating optical properties we cannot exclude any of the discussed mechanisms with certainty what is the physical basis for the excitation dependent emission. It would be helpful to examine the optical properties on the single cluster level or in low concentrated solution to excludes collective effects and a potential crystal-site heterogeneity as a main cause. Yet due to the purity of the single crystals and the diffraction data we are confident that crystal-site heterogeneity has a limited effect.

# 7. References


[1] E. Zeller, H. Beruda, H. Schmidbaur, Tetrahedral gold cluster $[Au_4]^{2+}$: crystal structure of $[[(\textit{tert}\text{-}Bu)_3PAu]_4]^{2+}(BF_4^-)_2.2CHCl_3$, *Inorganic Chemistry* **1993**, 32, 3203, 10.1021/ic00067a002.

[2] F. Demartin, M. Manassero, L. Naldini, R. Ruggeri, M. Sansoni, Synthesis and X-ray characterization of an iodine-bridged tetranuclear gold cluster, di-µ-iodo-tetrakis(triphenylphosphine)-tetrahedro-tetragold, *Journal of the Chemical Society, Chemical Communications* **1981**, 222, 10.1039/C39810000222.

[3] T. M. Dau, Y.-A. Chen, A. J. Karttunen, E. V. Grachova, S. P. Tunik, K.-T. Lin, W.-Y. Hung, P.-T. Chou, T. A. Pakkanen, I. O. Koshevoy, Tetragold(I) Complexes: Solution Isomerization and Tunable Solid-State Luminescence, *Inorganic Chemistry* **2014**, 53, 12720, 10.1021/ic501470v.

[4] D. Li, C.-M. Che, S.-M. Peng, S.-T. Liu, Z.-Y. Zhou, T. C. W. Mak, Spectroscopic properties and crystal structures of luminescent linear tri- and tetra-nuclear gold(I) complexes with bis(diphenylphosphinomethyl)phenylphosphine ligand, *Journal of the Chemical Society, Dalton Transactions* **1993**, 189, 10.1039/DT9930000189.

[5] S. G. Balasubramani, G. P. Chen, S. Coriani, M. Diedenhofen, M. S. Frank, Y. J. Franzke, F. Furche, R. Grotjahn, M. E. Harding, C. Hättig, A. Hellweg, B. Helmich-Paris, C. Holzer, U. Huniar, M. Kaupp, A. Marefat Khah, S. Karbalaei Khani, T. Müller, F. Mack, B. D. Nguyen, S. M. Parker, E. Perlt, D. Rappoport, K. Reiter, S. Roy, M. Rückert, G. Schmitz, M. Sierka, E. Tapavicza, D. P. Tew, C. van Wüllen, V. K. Voora, F. Weigend, A. Wodyński, J. M. Yu, TURBOMOLE: Modular program suite for ab initio quantum-chemical and condensed-matter simulations, *Journal of Chemical Physics* **2020**, 152, 10.1063/5.0004635.

[6] C. Steffen, K. Thomas, U. Huniar, A. Hellweg, O. Rubner, A. Schroer, TmoleX—A graphical user interface for TURBOMOLE, *Journal of Computational Chemistry* **2010**, 31, 2967, 10.1002/jcc.21576.

[7] A. D. Becke, Density-functional exchange-energy approximation with correct asymptotic behavior, *Phys. Rev. A* **1988**, 38, 3098, 10.1103/PhysRevA.38.3098.

[8] J. P. Perdew, Density-functional approximation for the correlation energy of the inhomogeneous electron gas, *Physical Review B* **1986**, 33, 8822, 10.1103/PhysRevB.33.8822.

[9] C. Adamo, V. Barone, Toward reliable density functional methods without adjustable parameters: The PBE0 model, *Journal of Chemical Physics* **1999**, 110, 6158, 10.1063/1.478522.

[10] E. Caldeweyher, C. Bannwarth, S. Grimme, Extension of the D3 dispersion coefficient model, *Journal of Chemical Physics* **2017**, 147, 10.1063/1.4993215.

[11] E. Caldeweyher, S. Ehlert, A. Hansen, H. Neugebauer, S. Spicher, C. Bannwarth, S. Grimme, A generally applicable atomic-charge dependent London dispersion correction, *Journal of Chemical Physics* **2019**, 150, 10.1063/1.5090222.

[12] E. Caldeweyher, J.-M. Mewes, S. Ehlert, S. Grimme, Extension and evaluation of the D4 London-dispersion model for periodic systems, *Physical Chemistry Chemical Physics* **2020**, 22, 8499, 10.1039/D0CP00502A.

[13] F. Weigend, R. Ahlrichs, Balanced basis sets of split valence, triple zeta valence and quadruple zeta valence quality for H to Rn: Design and assessment of accuracy, *Phys. Chem. Chem. Phys.* **2005**, 7, 3297, 10.1039/B508541A.

[14] F. Weigend, Accurate Coulomb-fitting basis sets for H to Rn, *Physical Chemistry Chemical Physics* **2006**, 8, 1057, 10.1039/B515623H.

[15] D. Andrae, U. Häußermann, M. Dolg, H. Stoll, H. Preuß, Energy-adjustedab initio pseudopotentials for the second and third row transition elements, *Theoretical Chemistry Accounts* **1990**, 77, 123, 10.1007/BF01114537.

[16] F. Neese, Software update: The ORCA program system—Version 5.0, *WIREs Comput Mol Sci* **2022**, 12, e1606, 10.1002/wcms.1606.

[17] F. Neese, An improvement of the resolution of the identity approximation for the formation of the Coulomb matrix, *Journal of Computational Chemistry* **2003**, 24, 1740, 10.1002/jcc.10318.

[18] F. Neese, F. Wennmohs, A. Hansen, U. Becker, Efficient, approximate and parallel Hartree–Fock and hybrid DFT calculations. A 'chain-of-spheres' algorithm for the Hartree–Fock exchange, *Chemical Physics* **2009**, 356, 98, 10.1016/j.chemphys.2008.10.036.
[19] F. Neese, The SHARK integral generation and digestion system, *Journal of Computational Chemistry* **2023**, 44, 381, 10.1002/jcc.26942.
[20] B. Helmich-Paris, A trust-region augmented Hessian implementation for restricted and unrestricted Hartree–Fock and Kohn–Sham methods, *Journal of Chemical Physics* **2021**, 154, 10.1063/5.0040798.
[21] F. Neese, G. Olbrich, Efficient use of the resolution of the identity approximation in time-dependent density functional calculations with hybrid density functionals, *Chemical Physics Letters* **2002**, 362, 170, 10.1016/S0009-2614(02)01053-9.
[22] D. Peng, N. Middendorf, F. Weigend, M. Reiher, An efficient implementation of two-component relativistic exact-decoupling methods for large molecules, *Journal of Chemical Physics* **2013**, 138, 10.1063/1.4803693.
[23] L. Cheng, J. Gauss, Analytic second derivatives for the spin-free exact two-component theory, *Journal of Chemical Physics* **2011**, 135, 10.1063/1.3667202.
[24] L. Visscher, K. G. Dyall, DIRAC–FOCK ATOMIC ELECTRONIC STRUCTURE CALCULATIONS USING DIFFERENT NUCLEAR CHARGE DISTRIBUTIONS, *Atomic Data and Nuclear Data Tables* **1997**, 67, 207, 10.1006/adnd.1997.0751.
[25] P. Pollak, F. Weigend, Segmented Contracted Error-Consistent Basis Sets of Double- and Triple-ζ Valence Quality for One- and Two-Component Relativistic All-Electron Calculations, *Journal of Chemical Theory and Computation* **2017**, 13, 3696, 10.1021/acs.jctc.7b00593.
[26] Y. J. Franzke, R. Treß, T. M. Pazdera, F. Weigend, Error-consistent segmented contracted all-electron relativistic basis sets of double- and triple-zeta quality for NMR shielding constants, *Physical Chemistry Chemical Physics* **2019**, 21, 16658, 10.1039/C9CP02382H.
[27] F. Neese, Efficient and accurate approximations to the molecular spin-orbit coupling operator and their use in molecular g-tensor calculations, *Journal of Chemical Physics* **2005**, 122, 10.1063/1.1829047.
[28] W. R. Dawson, M. W. Windsor, Fluorescence yields of aromatic compounds, *Journal of Physical Chemistry* **1968**, 72, 3251, 10.1021/j100855a027.
[29] W. H. Melhuish, QUANTUM EFFICIENCIES OF FLUORESCENCE OF ORGANIC SUBSTANCES: EFFECT OF SOLVENT AND CONCENTRATION OF THE FLUORESCENT SOLUTE, *Journal of Physical Chemistry* **1961**, 65, 229, 10.1021/j100820a009.
[30] K. Suzuki, A. Kobayashi, S. Kaneko, K. Takehira, T. Yoshihara, H. Ishida, Y. Shiina, S. Oishi, S. Tobita, Reevaluation of absolute luminescence quantum yields of standard solutions using a spectrometer with an integrating sphere and a back-thinned CCD detector, *Phys. Chem. Chem. Phys.* **2009**, 11, 9850, 10.1039/B912178A.
[31] G. Sheldrick, Crystal structure refinement with SHELXL, *Acta Crystallographica Section C* **2015**, 71, 3, 10.1107/S2053229614024218.
[32] G. M. Sheldrick, A short history of SHELX, *Acta Crystallographica Section A* **2008**, 64, 112, 10.1107/s0108767307043930.
[33] O. V. Dolomanov, L. J. Bourhis, R. J. Gildea, J. A. K. Howard, H. Puschmann, OLEX2: a complete structure solution, refinement and analysis program, *Journal of Applied Crystallography* **2009**, 42, 339, 10.1107/S0021889808042726.
[34] J. C. del Valle, J. Catalán, Kasha's rule: a reappraisal, *Physical Chemistry Chemical Physics* **2019**, 21, 10061, 10.1039/c9cp00739c.
[35] O. Racchi, S. Scurti, S. Liotino, A. Lanfranchi, A. Painelli, D. Comoretto, N. Došlić, D. Caretti, C. Sissa, Freezing Out Vavilov's Rule: Temperature-Dependent Fluorescence of a Rhodanine Dye, *ChemPhotoChem* **2026**, 10, e202500255, 10.1002/cptc.202500255.
[36] T. D. Green, C. Yi, C. Zeng, R. Jin, S. McGill, K. L. Knappenberger, Jr., Temperature-Dependent Photoluminescence of Structurally-Precise Quantum-Confined $Au_{25}(SC_8H_9)_{18}$ and $Au_{38}(SC_{12}H_{25})_{24}$ Metal Nanoparticles, *The Journal of Physical Chemistry A* **2014**, 118, 10611, 10.1021/jp505913j.

[37] L. Luo, Z. Liu, X. Du, R. Jin, Photoluminescence of the $Au_{38}(SR)_{26}$ nanocluster comprises three radiative processes, *Communications Chemistry* **2023**, 6, 22, 10.1038/s42004-023-00819-3.
[38] G. Marineau-Plante, F. Juvenal, A. Langlois, D. Fortin, A. Soldera, P. D. Harvey, Multiply "trapped" $^3$[*trans*-$Pt(PR_3)_2(C{\equiv}CC_6H_4X)_2$]* conformers in rigid media, *Chemical Communications* **2018**, 54, 976, 10.1039/c7cc09503a.
[39] I.-F. Hung, T. Okamura, S. Sugamori, R. W. Yip, Excitation Wavelength Dependence of Quinoline Phosphorescence Lifetime, *Bulletin of the Chemical Society of Japan* **1978**, 51, 3383, 10.1246/bcsj.51.3383.
[40] H. Schmidbaur, H. G. Raubenheimer, Excimer and Exciplex Formation in Gold(I) Complexes Preconditioned by Aurophilic Interactions, *Angewandte Chemie, Internationale Edition* **2020**, 59, 14748, 10.1002/anie.201916255.
[41] A. J. Blake, R. Donamaría, V. Lippolis, J. M. López-de-Luzuriaga, M. Monge, M. E. Olmos, A. Seal, J. A. Weinstein, Unequivocal Experimental Evidence of the Relationship between Emission Energies and Aurophilic Interactions, *Inorganic Chemistry* **2019**, 58, 4954, 10.1021/acs.inorgchem.8b03621.
